# Self-reported experiences of interpersonal racial discrimination and maternal and neonatal health: a systematic review and meta-analysis

**DOI:** 10.3389/frph.2026.1783126

**Published:** 2026-03-16

**Authors:** Maryam Adesunkanmi, Shi Jie (Angel) Zhou, Huda F. Al-Shamali, Sana Amjad, Tona M. Pitt, Oluwabukola Salami, Jesus Serrano-Lomelin, Maria B. Ospina

**Affiliations:** 1 Department of Obstetrics and Gynecology, Faculty of Medicine and Dentistry, University of Alberta, Edmonton, AB, Canada; 2 Department of Public Health Sciences, Faculty of Health Sciences, Queen’s University, Kingston, ON, Canada; 3 Interventional Psychiatry Program, St. Michael’s Hospital, Toronto, ON, Canada; 4 Department of Pediatrics, Cumming School of Medicine, University of Calgary, Calgary, AB, Canada; 5 Department of Community Health Sciences, Cumming School of Medicine, University of Calgary, Calgary, AB, Canada

**Keywords:** infant, maternal health, meta-analysis, pregnancy, pregnancy outcome, racism, social discrimination, systematic review

## Abstract

**Introduction:**

Racial discrimination contributes to maternal and neonatal health inequities. We synthesized evidence on associations between self-reported interpersonal racial discrimination and maternal and neonatal outcomes.

**Methods:**

We searched six major bibliographic databases from inception to September 2024, updated October 2025. We included observational epidemiological studies with comparison groups among pregnant or previously pregnant women. Outcomes included hypertensive disorders of pregnancy (HDP), gestational diabetes mellitus (GDM), mode of delivery, postpartum depression (PPD), fetal growth and gestational outcomes, infant mortality, and neonatal intensive care unit (*N*ICU) admission. Two independent reviewers screened studies and assessed risk of bias using the Newcastle-Ottawa Scales and the Appraisal Tool for Cross-Sectional Studies. Random-effects meta-analyses generated pooled adjusted odds ratios (aOR) with 95% confidence intervals (CI).

**Results:**

From 20,361 records, 61 publications of 63 studies including 1,473,417 participants were included. No associations were reported for HDP or GDM. Evidence was strongest for PPD, with higher odds in cohort (pooled aOR 1·37, 95% CI 1·16–1·63) and cross-sectional studies (pooled aOR 1·82, 95% CI 1·35–2·47). Cohort studies showed no association with PTB, whereas cross-sectional studies indicated increased odds (pooled aOR 1·19, 95% CI 1·03–1·38). Higher odds were observed for low birth weight (LBW) (pooled aOR 2·21, 95% CI 1·46–3·35), and very LBW (pooled aOR 2·70, 95% CI 1·40–5·20). Evidence for other outcomes was inconsistent. No studies examined infant mortality or NICU admission. Most included studies were at moderate risk of bias.

**Conclusions:**

Interpersonal racial discrimination is associated with PPD and LBW. Racial discrimination should be considered a modifiable determinant of maternal and neonatal health and integrated into perinatal research and care to reduce inequities.

**Systematic Review Registration:**

https://www.crd.york.ac.uk/PROSPERO/view/CRD42022312529, identifier CRD42022312529.

## 1 Introduction

Persistent racial and ethnic disparities in maternal and neonatal outcomes remain a major global concern, particularly in high-income and upper-middle-income countries, where most evidence is available (1). Across these settings, racialized populations (including Black and Indigenous peoples, and other groups minoritized through processes of racialization) experience higher risks of adverse perinatal outcomes, including preterm birth (PTB), low birthweight (LBW), unplanned cesarean delivery, and postpartum depression (PPD), compared with more socially advantaged groups (2, 3). Although social determinants such as socioeconomic position, educational attainment, and neighborhood deprivation account for part of these inequities (4), additional pathways are likely to contribute, including psychosocial stressors rooted in racism.

Racial discrimination is not confined to any single population and varies across social and historical context, with patterns linked to colonization, migration, and systems of power that racialize different groups across settings. Racial discrimination refers to unfair treatment based on race, ethnicity (often racialized through markers such as skin color, or national origin) and operates through both interpersonal and structural mechanisms (5). Self-reported interpersonal discrimination captures individual-level exposure to racism in everyday interactions and has been associated with immune, inflammatory, and vascular dysregulation relevant to adverse perinatal outcomes (6, 7). In contrast, structural indicators, such as racialized economic segregation and neighbourhood privilege, reflect area-level inequities but do not account for interpersonal forms of racism that may independently influence maternal and neonatal health (8).

The weathering hypothesis provides a framework for interpreting these associations, proposing that chronic exposure to racism and structural disadvantage accelerates physiological wear and biological aging, increasing susceptibility to adverse perinatal outcomes (9). Evidence consistent with weathering has been reported across Black, Indigenous peoples, and other racialized populations in different global settings (10).

Previous reviews have examined interpersonal racial discrimination in relation to selected perinatal outcomes, such as PTB, LBW, and specific obstetric complications (11–16), while others have focused on structural forms of racism (17, 18). Most have addressed a limited group of outcomes, specific forms of racism, or particular populations. This systematic review expands the current evidence base by synthesizing the literature across a broader spectrum of maternal and neonatal outcomes related to self-reported interpersonal racial discrimination.

## 2 Methods

This systematic review and meta-analysis followed the Preferred Reporting Items for Systematic review and Meta-Analysis (PRISMA) 2020 guidelines (19). The review protocol was registered with the International Prospective Register of Systematic Reviews (Protocol CRD42022312529).

### 2.1 Search strategy and selection criteria

We systematically searched MEDLINE (Ovid), EMBASE (Ovid), APA PsycINFO (Ovid), SCOPUS, and CINAHL Plus (EBSCOhost) from database inception to September 2024, with an updated search in October 2025. Grey literature was identified through ProQuest Theses and Dissertations, Google Scholar, and reference lists of relevant studies. No language restrictions were applied. Search terms combined concepts related to pregnancy, racism, discrimination, and maternal or neonatal outcomes. The MEDLINE search strategy is available in Supplementary Table S1.

Eligible studies were observational epidemiological designs (retrospective or prospective cohort, case-control, or cross-sectional) involving pregnant or previously pregnant women that compared groups with vs. without self-reported individual-level experiences of interpersonal racial discrimination, measured distinctly from other forms of discrimination, and regardless of age, pregnancy trimester, or ethnicity. Eligible maternal outcomes were hypertensive disorders of pregnancy (HDP), gestational diabetes mellitus (GDM), mode of delivery, and PPD. Neonatal outcomes included gestational outcomes (i.e., PTB), and fetal growth outcomes [LBW, small for gestational age (SGA), and large for gestational age (LGA)], infant mortality, and admission to a neonatal intensive care unit (NICU). These key outcomes were selected for their established links to adverse maternal and neonatal health (20). We excluded studies that used only aggregate or area-level indicators of racial discrimination (e.g., neighbourhood-level or segregation measures) or in which interpersonal racial discrimination could not be distinguished from other forms of discrimination. Editorials, commentaries, and review articles were also excluded. When conference abstracts were identified, we searched for a corresponding full-text publication; if none was found, the abstract was excluded. Records were managed in Covidence. Two reviewers (MA or MO, and one of SJZ, HA, SA, JM, or TP) independently screened titles, abstracts, and full-text articles. Disagreements were resolved through discussion and consensus.

### 2.2 Data extraction and risk of bias assessment

Data on study characteristics, population, exposure measurement, outcomes, measures of association, and covariates included in adjusted models were extracted into a standardized form by one reviewer (HA, SJZ, SA, JM, MBO, or TMP) and independently verified for accuracy by a second reviewer (MA, MBO).

Risk of bias for cohort and case-control studies were assessed independently by two reviewers (MA or MBO, and one of SJZ, HA, SA, JM, or TMP) using the Newcastle-Ottawa Scales (NOS), which evaluates selection, comparability, and outcome (or exposure) domains (21). The NOS uses a star system, with values ranging from 0 to 9 to summarize study quality. Overall risk of bias was classified as low (8–9 stars), moderate (6–7 stars), and high risk (≤5 stars). Domain-specific thresholds were: Selection (4 = low, 2–3 = moderate, 0–1 high), Comparability (2 = low, 1 = moderate, 0 = high), and Exposure/Outcome (3 = low, 1–2 moderate, 0 = high). Cross-sectional studies were assessed using the 20-item Appraisal tool for Cross-Sectional Studies (AXIS) tool, rated as “Yes”, “No”, or “Unclear” (22). Items were grouped into five conceptual domains reflecting study design (items 1–3), sampling (items 4–7), measurement (items 8–10, 13), reporting (items 11, 12, 14, 15) and conflicts of interest (items 16–20) and rated as “Yes”, “No”, or “Unclear”. For each domain, the proportion of items rated “Yes” was calculated, with ≥75% indicating low risk of bias, 50%–74% moderate, and <50% high risk. As the AXIS tool does not specify a standardized method for overall appraisal, and overall risk of bias classification was derived by averaging the five domain-level proportions using the same thresholds for low, moderate, and high risk. Discrepancies in risk of bias assessments were resolved through consensus. Risk of bias judgements were summarized using traffic-light plots and weighted bar graphs prepared in *Robvis*, showing the proportion of low, moderate, and high risk of bias ratings across studies and domains. Data extraction and risk of bias assessments were managed using Microsoft Excel.

### 2.3 Data analysis and synthesis of results

For outcomes where meta-analysis was not feasible, study findings were synthesized following the Synthesis Without Meta-analysis (SWiM) reporting guidelines (23). Evidence tables and evidence direction plots were prepared to summarize study characteristics and findings. When studies did not provide a measure of association, unadjusted odds ratios (OR) or risk ratios (RR) and their 95% confidence intervals (CI) were calculated from reported cell counts or percentages. When adjusted estimates were presented separately for subgroups (e.g., by race or ethnicity), log-transformed adjusted OR (aOR) and their standard errors were pooled using inverse-variance weighting to obtain a single-study-level estimate. In studies reporting regression coefficients rather than ORs, unstandardized logistic regression β coefficients (log-odds scale) and their 95% CIs were exponentiated to derive corresponding ORs. Standardized coefficients were not pooled. Meta-analyses were conducted for outcomes with at least two studies that were clinically and methodologically comparable. To minimize confounding, we pooled only fully adjusted measures of association from models that accounted for key confounders, stratified by study design and outcome (24). When studies reported subgroup-specific estimates without an overall pooled estimate, we selected the subgroup most comparable to the populations included in the other studies. For dichotomous outcomes, pooled adjusted RR (aRR) or OR (aOR) with 95% CI were calculated using the inverse-variance method under a random-effects model. Between-study heterogeneity was quantified using the I^2^ statistic and categorized as low (<25%), moderate (26%–74%), or high (≥75%). Results were displayed in forest plots. Formal sensitivity analyses were not feasible due to the small number of studies per outcome; we therefore assessed robustness through design-stratified analyses where possible. All statistical analyses were conducted in Stata (release 18; StataCorp, College Station, TX, USA). Publication bias assessment was planned when at least ten studies contributed to the same pooled meta-analysis.

## 3 Results

**Figure 1:**
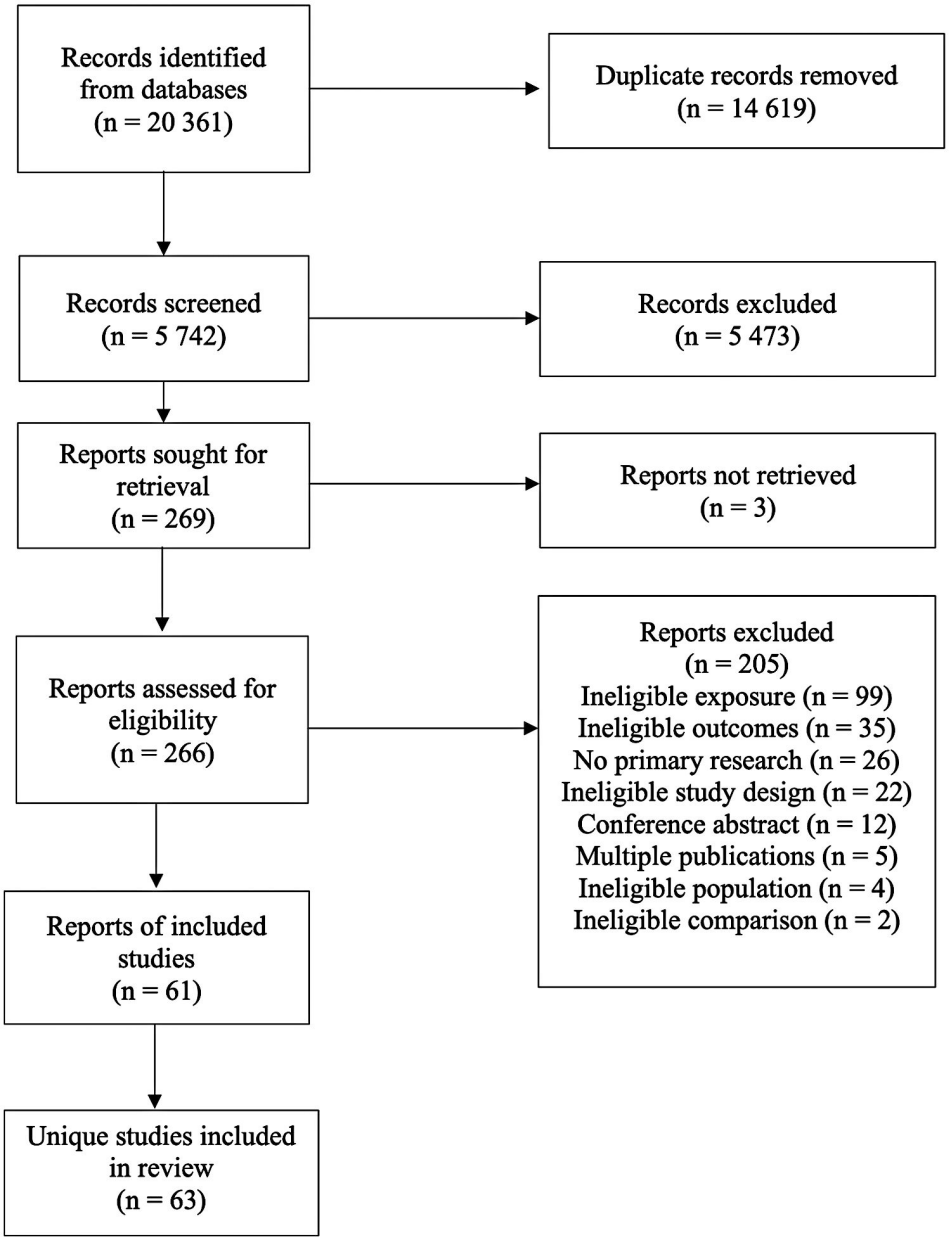
PRISMA flow diagram for study selection.

The literature searches identified 20,361 records. After removing duplicates, 5,742 titles and abstracts were screened, and 269 full-text articles were assessed for eligibility. Three were under embargo at the time of the review, leaving 266 full texts reviewed. Sixty-one reports (25–85) were included, two publications (49, 65) each reported findings from two distinct studies analyzed separately yielding to 63 independent studies included in the review (Figure 1). The full references of the 205 excluded studies are available upon request.

The 63 studies, published between 1991 and 2025, included 1,473,417 participants aged 15 years or older, with a mean age of approximately 27·5 years across studies. Most were conducted in the USA (*n* = 58) (25–28, 30, 31, 33–60, 62–73, 75–81, 83–85), and included samples that were either entirely composed of African American or Black participants (*n* = 26) (26, 31, 35–40, 47, 49, 51, 54–56, 58–60, 64, 68, 70, 71, 73, 79, 80, 84) or predominantly non-Hispanic Black within racially diverse study populations (*n* = 9) (25, 41, 53, 66, 67, 72, 76, 78, 85). Three studies focused exclusively on Hispanic or Latina women (33, 65), while others (*n* = 20) (27, 28, 30, 34, 42–46, 48, 50, 52, 57, 62, 63, 69, 75, 77, 81, 83) included racially and ethnically diverse samples. Two studies (29, 82) conducted in Aotearoa New Zealand examined racial discrimination among Māori, Pacific, and Asian women, one study (32) assessed Aboriginal and Torres Strait Islander women in South Australia, one (61) in Serbia and Macedonia examined Roma women, and one (74) in Germany focused on Turkish immigrants. Participants included pregnant (*n* = 28) (25, 26, 28, 29, 34–36, 40, 41, 43–45, 47, 49, 54, 56–58, 60, 62, 65, 67, 70, 71, 73, 82), postpartum (*n* = 29), (27, 30–33, 37, 38, 42, 46, 48, 51–53, 55, 59, 61, 63, 64, 66, 72, 74, 76–79, 81, 83–85) and mixed samples of pregnant and postpartum women (*n* = 2) (68, 80). A few studies (*n* = 4) (39, 50, 69, 75) included women with prior pregnancy history, regardless of whether they were pregnant or postpartum at the time of assessment.

Study designs comprised 32 cohort [25 prospective (25, 28, 29, 34, 35, 41, 43–45, 47, 49, 54, 56–58, 60, 62, 65, 69, 70, 73, 79, 82), four retrospective, (53, 67, 74, 76) and three ambi-directiona l^40^, ^68^, ^80^], 27 cross-sectional (26, 27, 30–33, 36, 39, 42, 46, 48, 50–52, 55, 59, 61, 63, 66, 72, 75, 77, 78, 81, 83–85), and four case-control studies (37, 38, 64) (Table 1).

**Table 1:** Summary of study characteristics and main findings from included studies.

Study, Country	Study Design	Population Characteristics	Exposure/Racial Discrimination Measure	Study Outcomes	Main Findings and Direction of Association**	Adjustment
						(Model and Covariates)
Ard et al., (26)	CSS	*N* = 107 pregnant women Ethnicity: Black (100%) Estimated* mean age = 39·8 yr, SD: 7·3	Maternal racial and discrimination stress	PTB (<37 wks)	PTB ▴: *r* = −0·31	N/A
USA			Jackson, Hogue, and Phillips Contextualized Stress Measure (Racial burden subscale score)			
Barber et al., (27)	CSS	*N* = 2,634 postpartum women Ethnicity: NHW (57·7%), NHB (20·5%), Hispanic other race (13·9%), and non-Hispanic other race (7·9%) Estimated* mean age: 29·8 yr, SD: 6·2	Past year experiences of discrimination or harassment due to race/ethnicity	PTB (<37 wks) LBW (<2,500 gr) SGA (<10th percentile)	PTB ◄► aOR 1·76, 95% CI 0·90–3·43 (all women); ▴aOR 7·18, 95% CI 2·28–22·65 (NHB women)	Multivariate logistic regression
						Race/ethnicity, age, education, annual household income, health insurance type, previous PTB, drinking/smoking during pregnancy, gestational diabetes
			One of two questions from the PRAMS questionnaire			
					LBW ▴ aOR 2·27, 95% CI 1·18–4·38 (all women); ▴aOR 3·56, 95% CI 1·28–9·91 (NHB women)	
USA						
					SGA ◄► aOR 1·31, 95% CI 0·70–2·45 (all women); ◄► aOR 1·95, 95% CI 0·55–6·87 (NHB women)	
Barcelona et al., (28)	PCS	*N* = 9,148 pregnant women Ethnicity: NHW (57·3%), NHB (11·8%), Hispanic (15·6%), Other (15·2%) Mean age = 26·9 yr, SD: 5·6	Individual experiences of racism and discrimination	Gestational age (weeks)	Gestational age (weeks) ◄► a*β* −0·10, 95% CI −0·29 to 0·09	Linear-mixed effects model
						Age, education, smoking status, gestational hypertension, preeclampsia, gestational diabetes
			Experiences of Discrimination scale (EOD) score			
USA						
Becares et al., (29)	PCS	*N* = 6,605 pregnant women Ethnicity: Māori (19·1%), Pacific Islander (15·6%), Asian (15·9%), European (49·4%) Mean age = 31·5 yr; SD: 5·3	Lifetime and past year experiences of racial discrimination	PPD (EPDS)	PPD ▴ aOR 1·49, 95% CI 1·08–2·05 (one experience; ever); ◄►aOR 1·37, 95% CI 0·95–1·97 (one experience; past 12 months); ▴ aOR 1·51, 95% CI 1·08–2·11 (≥2 experiences; ever); ◄►aOR 0·90, 95% CI 0·52–1·57 (≥2 experiences; past 12 months) (Māori, Pacific, and Asian women)	Multivariable logistic regression
						Age, ethnicity, household income, education, relationship status, area-level deprivation
Aotearoa New Zealand			Growing Up in New Zealand (GUiNZ) antenatal questionnaire			
Bossick et al., (30)	CSS	*N* = 632,387 postpartum women Ethnicity: Hispanic (37·4%), non-Hispanic (62·6%) Estimated* mean age: 29·3 yr; SD: 5·5	Past year experiences of emotional upset due to racism	PPD (PHQ-2)	PPD ▴ + 10·3 percentage points, 95% CI 6·8–13·8 (all PPOC); +13·6 points, 95% CI 8·8–18·5 (non-Hispanic PPOC); +4·1 points, 95% CI 1·4–8·0 (Hispanic PPOC)	Multivariable logistic regression; recycled predictions for adjusted probabilities
USA						
						Age, education, timely prenatal care, insurance type, stress during pregnancy, pre-pregnancy depression
			One question from the PRAMS questionnaire			
Bower et al., (31)	CSS	*N* = 11,582 postpartum women Ethnicity: NHB (100%) Estimated* mean age =25·8 yr, SD: 5·5	Past year experiences of emotional upset due to racism	PTB (<37 wks)	PTB ▴aOR 1·29, 95% CI 1·04–1·59	Multivariable logistic regression
						Age, education, marital status, smoking during pregnancy, pre-pregnancy BMI, insurance type
			One question from the PRAMS questionnaire			
USA						
Brown et al., (32)	CSS	*N* = 322 postpartum women Ethnicity: Aboriginal (100%) Mean age = 25·5 yr, SD: 5·6	Perinatal care perceived racial discrimination	PTB (<37 wks) LBW (<2,500 gr) SGA (<10th percentile) LGA (≥90th centile)	PTB ◄► aOR	Multivariable logistic regression
					LBW ◄► aOR 2·00, 95% CI 1·00–3·90	
					SGA ◄► aOR 1·70, 95% CI 0·90–3·20	
					1·10, 95% CI 0·50–2·10	
					LGA ◄► aOR 0·90, 95% CI 0·40–2·20	Parity, smoking, cannabis use, exposure to stressful events and social health issues during pregnancy
Australia			Four items adapted from the Measures of Indigenous Racism Experience (MIRE)			
Cabezas et al., (33)	CSS	*N* = 1,360 postpartum women Ethnicity: Latinas (100%) Estimated* mean age =28·5 yr, SD: 5·4	Past year experiences of emotional upset due to racism	PPD (Two questions from the PRAMS questionnaire)	PPD ▴ aβ 0·76, SE 0·42; *p* < 0·05 (Spanish-speaking Latinas); aβ 0·89, SE 0·37; *p* < 0·05 (English-speaking Latinas)^†^	Bootstrap binary logistic regression
						Age, education, income, insurance type, history of depression, plurality, race, pregnancy intention
USA						
			One question from the PRAMS questionnaire			
Christian et al., (34)	PCS	*N* = 39 pregnant women Ethnicity: AA (48·7%), White (51·3%) Age = NR	Lifetime experiences of perceived racial discrimination and responses to discrimination	Birthweight (gr)	Birthweight ▾ *r* = –0·52; *p* = 0·03 (active responses to discrimination; AA women)	N/A
USA						
			Experiences of Discrimination scale (EOD) score			
Clarke et al., (35)	PCS	*N* = 304 pregnant women Ethnicity: AA (100%) Mean age = 24·9 yr, SD: 4·7	Experiences of gendered racism	PTB (<37 wks)	PTB ▴ AME 0·013, 95% CI 0·002 to 0·024; *p* = 0·02 (Racism subscale)	Multivariable logistic regression with average marginal effects
			Jackson Hogue Phillips Reduced Common Contextualized Stress Measure			Age, education, insurance type, marital status, pre-pregnancy BMI, parity, previous PTB, alcohol use, smoking, ACE
USA						
Clarke et al., (36)	CSS	*N* = 428 pregnant women Ethnicity: Black (100%) Mean age = 25·0 yr, SD: 4·8	Experiences of gendered racism	PPD (EDPS)	PPD ▴ *r* = 0·53, *p* < 0·0001; aβ 0·22, 95% CI 0·04 to 0·08, *p* < 0·0001	Multivariable linear regression
USA			Jackson Hogue Phillips Reduced Common Contextualized Stress Measure			Age, education
Collins et al., (37)	CCS	*N* = 85 postpartum women Ethnicity: AA (100%) Age = NR	Perception of exposure to racial discrimination	VLBW (<1,500 gr)	VLBW ◄► aOR 3·20, 95% CI 0·90–11·30 (≥1 pregnancy discrimination domains)	Multivariable logistic regression
						Age, parity, prenatal care, social support, smoking, alcohol, and drug use
USA						
			Experiences of Discrimination (EOD) scale			
Collins et al., (38)	CCS	*N* = 312 postpartum women Ethnicity: AA (100%) Estimated* mean age =23·4 yr, SD: 5·2	Lifetime and pregnancy exposure to interpersonal racial discrimination	PTB (<37 wks) and VLBW (<1,500 gr) combined	PTB and VLBW combined ▴ aOR 2·60, 95% CI 1·20–5·30 (≥3 lifetime discrimination domains); ◄► aOR 1·70, 95% CI 1·00–9·20 (1–2 lifetime discrimination domains)	Multivariable logistic regression
						Age, education, smoking
USA						
			Questions adapted from the Experiences of Discrimination (EOD) scale and the Perceived Racism Scale			
Daniels et al., (39)	CSS	*N* = 173 women Ethnicity: AA (100%) Age range = 30–50 yr	Direct and vicarious racial discrimination across adulthood, adolescence and childhood	Preterm labor (<37 wks)	Preterm labor◄► aOR 1·09, 95% CI 0·91–1·30 (adult direct); aOR 1·13, 95% CI 0·88–1·44 (adult vicarious); aOR 1·48, 95% CI 1·00–2·19 (adolescent direct); aOR 1·27, 95% CI 0·98–1·63 (adolescent vicarious); aOR 1·10, 95% CI 0·80–1·49 (childhood direct); aOR 1·45, 95% CI 1·01–2·09 (childhood vicarious)	Multivariable logistic regression
						Number of pregnancies, income, education, employment, marital status
USA						
			Everyday Discrimination Scale (EDS) and questions adapted from the Experiences of Discrimination (EOD) scale			
Davidson et al., (40)	ACS	*N* = 845 pregnant women Ethnicity: AA (100%) Mean age = 23·2 yr SD: 5·7	Overall racism index (lifetime)	PTB (<37 wks)	PTB ◄► OR^§^ 0·99, 95% CI 0·65, 1·52	N/A
USA			Racism and Lifetime Experience Scale (RaLES)-Brief questionnaire			
Dixon et al., (41)	PCS	*N* = 539 pregnant women Ethnicity: Black (54·5%), Hispanic (23·6%), Asian (20·4), Other (1·5%) Mean age = 30 yr, SD: 5·8	Lifetime experiences of racial discrimination	Birthweight (z score) PPD (EPDS)	Birthweight ▾ lower fetal growth: z-score β −0·25, 95% CI −0·45 to −0·04; β −0·70, 95% CI −1·13 to −0·26 (Hispanic)	N/A
USA			Modified version of Experiences of Discrimination (EOD) scale		PPD ◄► RR 1·86, 95% CI 0·76–4·55 (1–2 vs. 0 domains of racism); RR 2·02, 95% CI 0·84–4·83 (3 + vs. 0 domains of racism)	
Docherty et al., (42)	CSS	*N* = 9,554 postpartum women Ethnicity: White (82·3%), Black (2·2%), Asian/Pacific Islander (5·7%), American-Indian (1·1%), Mixed (4·9%), Other (3·8%) Estimated* mean age =29·1 yr, SD: 5·4	Healthcare discrimination due to race or skin color	PPD (Two questions from the PRAMS questionnaire)	PPD ◄► aOR 1·09, 95% CI 0·67–1·77	Multivariable logistic regression
						Race, age, education, pre-pregnancy depression, domestic violence, life stressors, financial assistance, food insecurity
			One question from the PRAMS questionnaire			
USA						
Dole et al., (43)	PCS	*N* = 1,962 pregnant women Ethnicity: AA (36%), White (57·8%), Other (6·2%) Estimated* mean age =25·6 yr, SD: 4·9	Perceived racial discrimination score	PTB (<37 wks)	PTB ◄► aRR 1·40, 95% CI 1·00–2·00	Log-linear model
						Parity, poverty index
			Experiences of Discrimination (EOD) scale			
USA						
Dole et al., (44)	PCS	*N* = 1,898 pregnant women Ethnicity: AA (38·2%), White (61·8%) Estimated* mean age =25·4 yr, SD: 4·9	Perceived racial discrimination in prenatal care	PTB (<37 wks)	PTB ▴ aRR 1·80, 95% CI 1·10–2·90 (AA women only)	Log-linear model
						Age, parity, education, marital status, income, pre-pregnancy BMI, prenatal care, bacterial vaginosis
USA						
			Adapted from the Experiences of Discrimination (EOD) scale			
Dominguez et al., (45)	PCS	*N* = 124 pregnant women Ethnicity: AA (41·1%), NHW (58·9%) Mean age = 30·2 yr, SD: 4·7	Racism exposure across general life domains (direct and vicarious)	Birthweight (gr) Gestational age (wks)	Birthweight ▾ aβ −0·17; *p* < 0·05; −39·59 g (lifetime perceived racism; AA women); aβ −0·25; *p* < 0·01 (childhood vicarious racism)	Hierarchical linear regression
						Medical risk, education, prenatal clinic type
USA					Gestational age ◄► data not reported	
			Adapted from the Experiences of Discrimination (EOD) scale			
Du et al., (46)	CSS	*N* = 3,319 postpartum women Ethnicity: Chinese (36%), Japanese (2%), Filipino (5·1%), Other; Asian/Pacific Islander (56·9%) Estimated* mean age =30·2 yr, SD: 5·6	Past year experiences of emotional upset due to racism	PPD (Two questions from the PRAMS questionnaire)	PPD ▴ aOR 3·37, 95% CI 2·00–5·68	Multivariable logistic regression
USA						Age, education, marital status, race, insurance type, mode of delivery, plurality, previous births, pregnancy intention, alcohol use, smoking, history of depression, infant gender
			One question from the PRAMS questionnaire			
Eatman et al., (47)	r	*N* = 297 pregnant women Ethnicity: AA/Black (100%) Estimated* mean age =25·3 yr, SD: 4·0	Lifetime experiences of racial discrimination	Gestational age (wks) Birthweight (z score) PTB (<37 wks) SGA (<10th percentile)	Gestational age z score▾ aβ −0·08, 95% CI −0·13 to −0·03	Multivariable linear regression
					Birthweight z score ◄►aβ −0·01, 95% CI −0·02 to 0·01	Age, education, insurance type, household income, early pregnancy BMI, parity, infant sex, substance use
USA			Experiences of Discrimination (EOD) scale			
					Birthweight (grams) ◄►aβ 3·13, 95% CI −5·49 to 11·75	
					PTB ◄► aOR 1·04, 95% CI 1·00–1·11	
					SGA ◄► aOR 1·02, 95% CI 0·98–1·06	
			Jackson Hogue Phillips Contextualized Stress Measure			
Erbetta, (48)	CSS	*N* = 4,084 postpartum women Ethnicity: White (29·2%), Black (21·1%), Hispanic (32·5%), Asian/PI/Other (17·2%) Estimated* mean age =29·7 yr, SD: 6·3	Past year experiences of emotional upset due to racism	GDM (PRAMS or birth certificate)	GDM ◄► RR 1·57, 95% CI 1·19–2·06; aRR 1·24, 95% CI 0·87–1·78	Multivariable logistic regression
						Education, marital status, employment, insurance type, prenatal care, previous PTB, pre-pregnancy BMI, pregnancy intention, HPD, smoking and alcohol use in pregnancy, poverty
USA						
			One question from the PRAMS questionnaire			
Ertel et al., (49)	PCS	Project ACCESS *N* = 526 pregnant women -Ethnicity: AA/Black (100%) -Estimated* mean age = 26·9 yr, SD: 6·1	Experiences of perceived racial discrimination and response to unfair treatment	PPD (EPDS)	PPD ◄► aOR 1·48, 95% CI 1·24–1·78	Multivariable logistic regression
USA						Age, race/ethnicity, marital status, household income, education, nativity, social support, ethnic identity
			Modified version of Experiences of Discrimination (EOD) scale			
Ertel et al., (49)	PCS	Project Viva *N* = 352 pregnant women Ethnicity: AA/Black (100%) Estimated* mean age = 27·8 yr, SD: 6·2	Experiences of perceived racial discrimination and response to unfair treatment	PPD (EPDS)	PPD ◄► aOR 1·13, 95% CI 0·93–1·38	Multivariable logistic regression
USA						Age, race/ethnicity, marital status, household income, education, nativity, social support, ethnic identity, pregnancy intention
			Modified version of Experiences of Discrimination (EOD) scale			
Flores-Rodriguez et al., (50)	CSS	*N* = 353 women -Ethnicity: Latinx (100%) -Mean age = NR·	Lifetime experiences of racial discrimination	PPD (NR)	PPD ▴ aOR 2·06, 95% CI 1·18–3·60	Multivariable logistic regression
						Insurance type, partner support, household income, education
USA						
			Questions from the Latina Breastfeeding Survey			
Floyd James et al., (51)	CSS	*N* = 231 postpartum women Ethnicity: Black (100%) Mean age = 31 yr, SD: 4·2	Past year frequency of interpersonal racism and self-appraisal	PPD (EPDS, PHQ-8, PHQ-15)	PPD ▴ EPDS-3 total score: aβ 1·36, SE 0·15; 95% CI 1·06 to 1·66; *p* < 0·001; PHQ-8 total score: aβ 3·26, SE 0·27; 95% CI 2·73 to 3·79; *p* < 0·001; PHQ-15 total score: aβ 0·77, SE 0·36; 95% CI 0·05 to 1·50; *p* = 0·035	Multivariable logistic regression
						Age, income, education, insurance type, mental health diagnosis history, birth type, miscarriage, parity, death of children, marital status
USA						
			Racism and Life Experience Scale—Daily Life Experiences subscale (RaLES-DLE)			
Fowlin et al., (52)	CSS	*N* = 1,194 postpartum women Ethnicity: NHB (12%), Hispanics (25·9%), NHW (52·6%), NH Other (9·5%) Mean age = 33 yr, SD: 5·7	Experiences of interpersonal and healthcare racial discrimination	PTB (<37 wks) LBW (<2,500 gr) SGA (<10th percentile)	PTB ◄►Health care OR 1·38, 95% CI 0·60–3·20; Interpersonal OR 1·30, 95% CI 0·67–2·52	N/A
USA					LBW ◄► Health care OR 1·38, 95% CI 0·60–3·20; Interpersonal OR 1·46, 95% CI 0·79–2·72	
			Modified questions from the PRAMS questionnaire		SGA ◄► Health care OR 1·38, 95% CI 0·60–3·20; Interpersonal OR 1·48, 95% CI 0·78–2·84	
Fryer et al., (53)	RCS	*N* = 1,732 postpartum women Ethnicity: NHB (66.6%), Latina (33.4%) Mean age = 24·7, SD: 5·1	Experiences of frequent discrimination attributed to race/ethnicity, ancestry, skin color, or language	PTB (<37 wks)	PTB ◄► HR 1·00, 95% CI 0·50–2·00 (NHB); ▴ HR 3·90, 95% CI 1·10–13·30 (NHB)	N/A
USA						
			Everyday Discrimination scale			
Gillespie et al., (54)	PCS	*N* = 91 pregnant women Ethnicity: NHB (100%) Mean age = 26 yr, SD: ∼1·5*	Lifetime experiences of racial discrimination	Gestational age (weeks) HDP (gestational hypertension, preeclampsia) Mode of delivery (induction, CS) GDM (NR)	Gestational age ◄► median 39·2 vs. 39·1 weeks; *p* = 0·451	N/A
USA					HDP ◄► gestational hypertension: RR^§^ 1·28, 95% CI 0·36–4·60; preeclampsia: RR^§^ 1·00, 95% CI 0·07–15·0	
			Experiences of Discrimination (EOD) scale			
					Mode of delivery (induction or CS) ◄► RR^§^ 1·03, 95% CI 0·28–3·85	
					GDM ◄► not estimated	
Giurgescu et al., (55)	CSS	*N* = 72 AA postpartum women Ethnicity: AA (100%) Mean age = 23·3 yr, SD: 5·3	Lifetime experiences of racial discrimination	PTB (<37 wks)	PTB ◄► aOR 0·81, 95% CI 0·50–1·32 (situation); aOR 1·10, 95% CI 0·93–1·31 (frequency)	Multivariable logistic regression
						Objective physical and social disorder, violent crime, perceived crime, perceived physical and social disorder
USA						
			Experiences of Discrimination (EOD) scale			
Green et al., (56)	PCS	*N* = 136 pregnant women Ethnicity: AA (100%) Mean age = 24·4 yr, SD: 5·2	Perceptions of racism score	LBW (<2,500 gr) Gestational age (wks)	LBW ◄► sr² = 0·0005; F(1, 127) = 0·08; *p* > 0·05	N/A
USA						
			Perceptions of Racism Scale		Gestational age ◄► sr² = 0·0003; F(1, 127) = 0·10; *p* > 0·05	
Grobman, (57)	PCS	*N* = 9,470 pregnant women Ethnicity: NHW (60·4%), NHB (13·8%), Hispanic (16·8%), Asian (4%), Other (5%) Age = NR	Perceived exposure to racism	PTB (<37 wks) HDP (antepartum gestational hypertension, antepartum, intrapartum, or postpartum preeclampsia or eclampsia SGA (<10th percentile)	PTB ◄► aOR 0·91, 95% CI 0·67–1·23	Multivariable logistic regression
USA			Experiences of Discrimination (EOD) scale		HDP ◄► aOR 0·81, 95% CI 0·62–1·06	Age, BMI, smoking, medical comorbidities, race/ethnicity, indicated psychosocial measure
					SGA ◄► aOR 1·01, 95% CI 0·78–1·31	
Harden et al., (58)	PCS	NN = 118 pregnant women Ethnicity: AA (100%) Mean age = 25·2 yr, SD: 4·6	Perceived racial discrimination during pregnancy	PPD (EPDS)	PPD ▴ EPDS score *r* = 0·31, *p* < 0·01	N/A
USA			Everyday Discrimination scale			
Heldreth et al., (59)	CSS	*N* = 1,349 postpartum women Ethnicity: AA (100%) Mean age = 24·1 yr, SD: 4·9	Direct and vicarious exposure to racial discrimination during childhood, current experiences of racism/discrimination	PPD (EPDS)	PPD ▴ aβ 0·72, 95% CI 0·06 to 1·37; *p* = 0·03 (childhood direct discrimination); aβ 0·85, 95% CI 0·14 to 1·54; *p* = 0·02 (childhood vicarious discrimination)	Multivariable logistic regression with mediation
USA						Education, history of depression, household income
			Adapted from Experiences of Discrimination (EOD) scale and Everyday Discrimination scale			
Hilmert et al., (60)	PCS	*N* = 42 pregnant women Ethnicity: AA (100%) Mean age = 28·7 yr, SD: 5·1	Direct and vicarious exposure to racial discrimination during childhood, current experiences of racism/discrimination	Birthweight (gr)	Birthweight ▾ aβ −0·26; *p* < 0·05 (adult personal racism); ◄► aβ −0·27; *p* < 0·10 (childhood indirect racism)	Multivariable linear regression
						Age, BMI, parity, income, education, length of gestation
USA						
			Adapted from Experiences of Discrimination (EOD) scale			
Janevic et al., (61)	CSS	*N* = 410 postpartum women Ethnicity: Roma (100%) Estimated* mean age = 24·7 yr, SD: 4·5	Interpersonal discrimination	LBW (<2,500 gr)	LBW ▴ aOR 2·50, 95% CI 1·00–5·90	Log-binomial regression
						Age, parity, years at current residence, household wealth, institutional discrimination
			Everyday Discrimination Scale			
Serbia and Macedonia						
Korte et al., (62)	PCS	*N* = 369 pregnant women Ethnicity: White (52%), Black (48%) Estimated* mean age = 24·9 yr, SD: 4·6	Experiences of racial discrimination	Gestational age (weeks) Birthweight (gr)	Gestational age ◄► aβ −3·50, 95% CI −8·10 to 1·20 (Black women only)	Multivariable linear regression
USA			Researcher-developed questionnaire		Birthweight ◄► aβ −0·04, 95% CI −0·21 to 0·12 (Black women only)	Income, employment, copying style
Lee et al., (63)	CSS	*N* = 9,907 postpartum women Ethnicity: Hispanic Non-White (8·4%), Hispanic White (9·8%), Non-Hispanic American Indian & Hawaiian (2·8%), Non-Hispanic Asian (8·2%), NHB (27·2%), Non-Hispanic non-White or mixed race (8·8%), NHW (34·8%) Estimated* mean age = 29·4 yr, SD: 5·2	Perceived experiences with racism and discrimination	HDP (diagnosis recorded in PRAMS)	HDP ◄► aOR 0·94, 95% CI 0·74–1·20	Multivariable logistic regression
USA						Age, parity, plurality, pre-pregnancy BMI, socio-economic status, race, ethnicity, chronic hypertension, pre-GDM, GDM
			One question from the PRAMS questionnaire			
Lespinasse et al., (64)	CCS	*N* = 312 postpartum women Ethnicity: AA (100%) Estimated* mean age = 24·7 yr, SD: 4·4	Perceived racial discrimination	VLBW (<1,500 gr)	VLBW ▴ OR 1·90, 95% CI 1·20–3·00 (one racism domain); OR 2·70, 95% CI 1·30–5·40 (three or more racism domains)	N/A
USA			Adapted from Experiences of Discrimination (EOD) scale			
Liu et al., (65)	PCS	*N* = 84 pregnant women Ethnicity: Latina/Hispanic (100%) Mean age = 27·7 yr, SD: 5·1	Everyday discrimination, major discrimination	Gestational age (wks) Birthweight (gr) PTB (<37 wks) -LBW (<2,500 gr)	Gestational age ◄► *r* = −0·09 (everyday discrimination), *p* ≤ 0·1; ▾ *r* = −0·25 (major discrimination subscale), *p* ≤ 0·05; ▾ aβ −0·91, SE 0·35, *p* = 0·01 (major discrimination subscale)	Hierarchical linear regression
USA						Sex, income-needs ratio, parental cohabitation, immigration status, education
			Everyday Discrimination scale			
					Birthweight ◄► *r* = −0·19 (everyday discrimination), *p* ≤ 0·1; ▾ *r* = −0·25 (major discrimination subscale), *p* ≤ 0·05; ▾ aβ −261·98, SE 103·10, *p* = 0·01 (major discrimination subscale)	
					PTB ◄► *r* = 0·05 (everyday discrimination), *p* ≤ 0·1; ▴ *r* = 0·27 (major discrimination subscale), *p* ≤ 0·05	
					LBW ◄► *r* = 0·19 (everyday discrimination), *p* = 0·08; ◄► *r* = 0·07 (major discrimination subscale), *p* ≤ 0·1	
iu et al., 2025 (65)	PCS	*N* = 102 pregnant women Ethnicity: Latina/Hispanic (100%) Mean age = 26·1 yr, SD: 5·1	Everyday discrimination, major discrimination	Gestational age (wks) Birthweight (gr) PTB (<37 wks) LBW (<2,500 gr)	Gestational age ◄► *r* = −0·16, *p* ≤ 0·1 (everyday discrimination); ◄► *r* = −0·14, *p* ≤ 0·1 (major discrimination subscale)	Hierarchical linear regression
USA						Sex, income-needs ratio, parental cohabitation, immigration status, education
					Birthweight ▾ *r* = −0·30, *p* ≤ 0·01 (everyday discrimination); ▾ *r* = −0·23, *p* ≤ 0·05 (major discrimination subscale); ▾ aβ −22·77, SE 7·46, *p* = 0·003 (everyday discrimination); ▾ aβ −102·23, SE 44·73, *p* = 0·02 (major discrimination subscale)	
			Everyday Discrimination scale			
					PTB (%) ▴ *r* = 0·28, *p* ≤ 0·01 (everyday discrimination); ▴ *r* = 0·22, *p* ≤ 0·05 (major discrimination subscale)	
					LBW (%) ▴ *r* = 0·39, *p* ≤ 0·01 (everyday discrimination); ▴ *r* = 0·31, *p* ≤ 0·01 (major discrimination subscale)	
McDonald et al., (66)	CSS	*N* = 2,452 postpartum women Ethnicity: NHB (59·3%), NHA (8·8%), Hispanic (23·9%), Other (8·0%) Estimated* mean age = 27·9 yr, SD: 6·5	Past year experiences of emotional upset due to racism	PPD (Two questions from the PRAMS questionnaire)	PPD ◄► aOR 1·41, 95% CI 0·99, 2·02	Multivariable logistic regression
						Age, race/ethnicity, education, marital status, insurance type, history of depression
USA						
			One question from the PRAMS questionnaire			
Mendez et al., (67)	RCS	*N* = 3,462 pregnant women Ethnicity: NHB (68·5%), NHW (8·7%), Hispanic (22·8%) Estimated* mean age = 24·6 yr, SD: 5·3	Everyday experiences of discrimination, and major experiences of discrimination	- PTB (<37 wks)	PTB ◄► aRR 1·10, 95% CI 1·00–1·20 (everyday discrimination, NHB); aRR 0·90, 95% CI 0·70–1·20 (everyday discrimination, NHW); aRR 0·90, 95% CI 0·60–1·20 (everyday discrimination, Hispanic); aRR 1·00, 95% CI 0·80–1·30 (major discrimination, NHB); aRR 1·20, 95% CI 0·90–1·70 (major discrimination, NHW); aRR 1·10, 95% CI 0·60–1·90 (major discrimination, Hispanic)	Log-binomial regression
						Age, race/ethnicity, income, education, marital status, smoking and alcohol use, previous livebirths, home ownership, time living in neighbourhood
USA						
			Everyday Discrimination Questionnaire and Major Experiences of Discrimination Questionnaire			
Misra et al., (68)	ACS	*N* = 832 pregnant and postpartum women Ethnicity: AA/Black (100%) Mean age = 23·1 yr, SD: 5·6	Lifetime exposure to racism	- PTB (<37 wks)	PTB ◄► HR 0·88, 95% CI 0·58–1·35	N/A
			Racism and Lifetime Experiences Scale (RaLES), RaLES Daily Life Experiences (RaLES-DLE) scale, Racism-Related Experiences (RRE) scale			
USA						
Mustillo et al., (69)	PCS	*N* = 352 women Ethnicity: Black (43·2%), White (56·8%) Mean age = 34·1 yr, SD: 3·4	Lifetime experiences of racial discrimination	PTB (<37 wks) -LBW (<2,500 gr)	PTB ▴ aOR 3·05, 95% CI 1·29–7·24 (≥3 vs. 0 experiences); ◄► aOR 2·05, 95% CI 0·93–4·50 (1–2 vs. 0 experiences)	Multivariable logistic regression
						Race, ethnicity, education, income, smoking, alcohol use, depressive symptoms, and gestational age (for LBW only)
					LBW ◄► aOR 1·06, 95% CI 0·29–3·84 (≥3 vs. 0 experiences); ◄► aOR 1·56, 95% CI 0·32–7·76 (1–2 vs. 0 experiences)	
USA						
			Experiences of Discrimination (EOD) scale			
Njoroge et al., (70)	PCS	*N* = 151 pregnant women Ethnicity: Black (100%) Mean age = 30·2 yr, SD: 5·7	Experiences of interpersonal racism	PPD (EPDS)	PPD ▴ aOR 1·60, 95% CI 1·07–2·39	Multivariable logistic regression
						Negative COVID-19 pandemic experience, systemic racism
			Everyday Discrimination Scale, Major Experiences of Discrimination Scale			
USA						
Rankin et al., (71)	CCS	*N* = 277 pregnant women Ethnicity: AA (100%) Estimated* mean age = 25·7 yr, SD: 5·4	Lifetime and past year interpersonal racial discrimination in public settings	PTB (<37 wks) and LBW (<2,500 gr) combined	PTB and LBW combined ▴ OR 1·50, 95% CI 0·90–2·80 (lifetime discrimination); OR 2·50, 95% CI 1·20–5·20 (past year discrimination)	N/A
USA						
			Modified version of the Perceived Racism Scale (PRS)			
Ringenary et al., (72)	CSS	*N* = 8,851 postpartum women Ethnicity: Black (43·9%), Hispanic (16·6%), Mixed race (11·4%), Asian (10·7%), Other Non-White (9·8%), Native American (7·6%) Estimated* mean age = 29·6 yr, SD: 6·0	Past year experiences of emotional upset due to racism	PPD (One question from the PRAMS questionnaire)	PPD ▴ aOR 2·38, 95% CI 1·19–3·38 (Black); ◄► aOR 1·08, 95% CI 0·83–1·80 (Hispanic); ▴ aOR 2·04, 95% CI 1·07–3·91 (Native American); ▴ aOR 2·51, 95% CI 1·28–4·93 (Other Non-White); ▴ aOR 2·80, 95% CI 1·49–5·27 (Mixed race); ▴ aOR 7·99, 95% CI 3·52–18·15 (Asian)^†^	Multivariable logistic regression
						Marital status, place of residence, pre-pregnancy depression, years of education
USA						
			One question from the PRAMS questionnaire			
					▴ pooled aOR 2·00, 95% CI 1·59–2·52 (all race groups)^‡^	
Rosenberg et al., (73)	PCS	*N* = 4,966 pregnant women Ethnicity: Black (100%) Estimated* mean age = 32·2 yr, SD: 4·5	Experiences of racial discrimination	PTB (<37 wks) LBW (<2,500 gr)	PTB ▴ aOR 1·30, 95% CI 1·10–1·60 (unfair treatment on the job); ◄► aOR 1·40, 95% CI 1·00–1·90 (people acting afraid monthly)	Generalized estimating equation logistic regression
			Researcher-developed questionnaire			Age, education, marital status, parity, previous PTB, maternal PTB, smoking
					LBW ◄► OR 1·40, 95% CI 0·80–2·20 (unfair treatment on the job); OR 1·40, 95% CI 0·60–3·30 (people acting afraid monthly); OR 1·20, 95% CI 0·60–2·60 (people acting afraid weekly)	
USA						
Scholaske et al., (74)	RCS	*N* = 2,525 postpartum women Ethnicity: German autochthonous (91·5%), Turkish Immigrants (8·6%) Mean age = 31·3 yr, SD: 5·4	Past year experiences of racial discrimination	PTB (<37 wks)	PTB ▴ aOR 5·76, 95% CI 1·95–19·38 (Turkish immigrant women)	Multivariable logistic regression
						Age, infant sex, education, parity, generation status
Germany						
			One question from the German Socio-Economic Panel survey			
Schuppie et al., (75)	CSS	*N* = 978 women Ethnicity: Black (23·8%), White (76·2%) Age = NR	Direct and vicarious racial discrimination over the life course	LBW (<2,500 gr)	LBW ◄► β −4·95; *p* = 0·96 (worry about current discrimination)	N/A
			Experiences of Discrimination (EOD) scale and researcher-developed questions			
USA						
Scroggins et al., (76)	RCS	*N* = 1,957 postpartum women Ethnicity: Black (69%), Hispanic (31%) Mean age = 24·6 yr, SD: 5·1	Typologies of lifetime discrimination experiences	PPD (EPDS)	PPD ▴ LSM 6·5, SE 0·3, *p* < 0·001(Typology 2 - high childhood–family discrimination; Black women); LSM 6·6, SE 0·5, *p* < 0·001 (Typology 3—moderate lifetime discrimination; Black women)	Latent class analysis; LSMs from linear mixed model.
						Education, employment, federal poverty category, insurance type, food insecurity, neighborhood problems
USA			Major Experiences of Discrimination Scale			
Segre et al., (77)	CSS	*N* = 2,805 postpartum women Ethnicity: NHW (35.4%), NHB (29%), Hispanic (35.6%) Estimated* mean age = 27·9 yr, SD: 5·4	Past year experiences of emotional upset due to racism	PPD (Two questions from the PRAMS questionnaire)	PPD ▴ aOR 2·15, 95% CI 1·07–4·31	Multivariable logistic regression
						Age, race/ethnicity, education, poverty level, marital status, history of depression
USA						
			One question from the PRAMS questionnaire			
Shour et al., (78)	CSS	*N* = 2,609 postpartum women Ethnicity: NHW (25.5%), NHB (49.5%), Hispanic (13.3%), Non-Hispanic Other (11.7%) Estimated* mean age = 28 yr, SD: 5·6	Past year experiences of emotional upset due to racism	PPD (One question from the PRAMS questionnaire)	PPD ◄► aOR 0·85, 95% CI 0·46–1·57	Multivariable logistic regression
						Age, race/ethnicity, education, marital status, poverty level, neighbor security, intimate partner violence, smoking, alcohol use, food security, insurance type, prenatal care, stressful events, BMI, disease conditions
USA			One question from the PRAMS questionnaire			
Slaughter-Acey et al., (79)	PCS	*N* = 1,232 postpartum women Ethnicity: AA/Black (100%) Estimated* mean age = 27·2 yr, SD: 5·8	Past year frequency and perceived stressfulness of racial micro-aggressions	PTB (<37 wks)	PTB ▴ OR 1·67, 95% CI 1·16–2·40 (moderate discrimination)	N/A
USA			Daily Life Experiences of Racism and Bother (DLE-B) scale			
Slaughter-Acey et al., (80)	ACS	*N* = 778 pregnant and postpartum women Ethnicity: AA (100%) Mean age = 23·3 yr, SD 5·7	Lifetime and past year personal experiences of racism, racial prejudice, or racial discrimination	SGA (<10th percentile)	SGA ◄► aOR 0·92, 95% CI 0·66–1·28 (≤18 years, overall racism); ◄► aOR 0·96, 95% CI 0·58–1·58 (≤18 years, personal racism)	Multivariable logistic regression
						Parity, education, employment, insurance type, height, household resources
					SGA ◄► aOR 0·86, 95% CI 0·69–1·06 (19–24 years, overall racism); ◄► aOR 0·86, 95% CI 0·64–1·18 (19–24 years, personal racism)	
			Adapted from the Racism and Lifetime Experience Scale (RaLES)-Brief questionnaire			
USA						
					SGA ▴ aOR 1·45, 95% CI 1·02–2·08 (≥25 years, overall racism); ◄► aOR 1·33, 95% CI 0·86–2·09 (≥25 years, personal racism)	
Spinner et al., (81)	CSS	*N* = 27,994 postpartum women Ethnicity: NHW (47·3%), NHB (19·9%), Hispanic (15·9%), Non-Hispanic (Other) (16·9%) Estimated* mean age = 28·7 yr, SD: 5·4	Past year experiences of emotional upset due to racism	Mode of delivery	◄► aOR 1·09, 95% CI 0·94–1·28 (CS)	Multivariable logistic regression
						Age, race/ethnicity, education, household income, insurance type, marital status, BMI, prenatal care, parity, previous PTB
USA						
			One question from the PRAMS questionnaire			
Thayer et al., (82)	PCS	*N* = 1,653 pregnant women Ethnicity: Māori (30.8%), Pacific (27.3%), Asian (41.8%) Mean age = 29·5 yr, SD: 5·6	Lifetime and past year experiences of discrimination attributed to ethnicity	Birthweight (gr) Gestational age (wks)	Birthweight (gr) ◄► lifetime ethnic discrimination: aβ −243, 95% CI −425 to −60 (work); aβ −146, 95% CI −286 to −6 (housing); (Māori women); aβ 188, 95% CI 7 to 369 (housing) (Asian women)	Multivariable linear regression
Aotearoa New Zealand						Age, BMI, household income, education, marital status, smoking, infant sex
			Growing Up in New Zealand (GUINZ) antenatal questionnaire			
					Gestational age (weeks) ▾aβ −1·06, 95% CI −1·83 to −0·28 (lifetime ethnicity-related physical attacks); aβ −0·95, 95% CI −1·56 to −0·34 (work), aβ −0·55 weeks, 95% CI −1·08 to −0·02 (justice); aβ −0·73 weeks, 95% CI −1·43 to −0·02 (banking) (Māori women)	
Thomas et al., (83)	CSS	*N* = 676,047 postpartum women Ethnicity: NHB (16.4%), NHW (52.2%), Hispanic (12.8%), Asian (7.4%), Indigenous (0.3%), Non-White Other (9.9%) Estimated* mean age = 27·6 yr, SD: 6·5	Past year experiences of emotional upset due to racism	PTB (<37 wks) -LBW (<2,500 gr) -Mode of delivery	PTB ▴ OR 1·49, 95% CI 1·45–1·53^§^	N/A
					LBW ▴ OR 1·59, 95% CI 1·54–1·64^§^	
					CS ▴ OR 1·45, 95% CI 1·42–1·48^§^	
USA						
			One question from the PRAMS questionnaire			
Walker et al., (84)	CSS	*N* = 67 postpartum women Ethnicity: AA (100%) Mean age: NR.	Experiences of gendered racism (total contextualized stress score)	PPD (EPDS)	PPD ◄► *r* = 0·20; *p* = 0·09	N/A
USA						
			Jackson, Hogue, Phillips Contextualized Stress Measure			
Weeks et al., (85)	CSS	*N* = 16,866 postpartum women Ethnicity: NHB (49.9%), Hispanic (36.5%), non-White other (23.1%) Estimated* mean age = 28·4 yr, SD: 5·9	Past year experiences of emotional upset due to racism	PPD (Two questions from the PRAMS questionnaire)	PPD ▴ aOR 2·70, 95% CI 2·20–3·40 (all women); aOR 3·50, 95% CI 2·60–4·80 (NHB women); aOR 2·20, 95% CI 1·4–3·4 (Hispanic), aOR 2·2, 95% CI 1·50–3·30 (NH women of other races)	Multivariable logistic regression
USA						Age, education, parity, smoking, insurance type, household poverty, pre-pregnancy BMI, stressful experiences
			One question from the PRAMS questionnaire			
Wheeler et al., (25)	PCS	*N* = 1,606 pregnant women Ethnicity: NHB (78.2%), NHW (21.8%) Mean age = 26·0 yr, SD: 6·1	Experiences of racism during current pregnancy and lifetime	PTB (<37 wks)	PTB ◄► aOR 1·06, 95% CI 0·90–1·25	Multivariable logistic regression
						Age, parity, race, chronic medical illness, chronic psychiatry history. tobacco use
USA						
			Perceived Racism Scale			

AA, African American; ACE, adverse childhood experiences; ACS, ambi-directional cohort study; AME, average marginal effect; aOR, adjusted odds ratio; aRR, adjusted risk ratio; AUC, area under curve; BMI, body mass index; CCS, case-control study; CS, caesarean section; CSS, cross-sectional study; EPDS, Edinburgh Depression Scale; EOD, experiences of discrimination; GDM, gestational diabetes mellitus; gr, grams; HDP, hypertensive disorders of pregnancy; HR, hazards ratio; LBW, low birth weight; LSM, least square mean; NHA, non-Hispanic Asian; NHB, non-Hispanic Black; NHW, non-Hispanic White; NR, not reported; OR, odds ratio; PCS, prospective cohort study; PHQ, Patient Health Questionnaire; PPD, postpartum depression; PPOC, postpartum people of color; PRAMS, Pregnancy Risk Assessment Monitoring System; PTB, preterm birth; RR, risk ratio; RCS, retrospective cohort study; RCT, randomized controlled trial; SD, standard deviation; SE, standard error; SGA, small for gestational age; sr², semi-partial correlation; VLBW, very low birthweight; wks, weeks; year(s), yr.

◄►: No significant findings or mixed results, ▴ = higher odds/risk of outcome; ▾ = lower odds/risk of outcome.

* Estimated mean (and SD) ages were calculated from categorical data using midpoints of reported age ranges weighted by group proportions.

** All measures reflect the association between higher self-reported racial discrimination and the specified perinatal outcome, unless otherwise indicated.

† Reported β coefficients were log-transformed and exponentiated to provide comparable aOR with 95% CI.

‡ Pooled aOR calculated from reported log-transformed coefficients.

§ Unadjusted measures of association calculated by review authors from reported cell counts in the publication.

The Experiences of Discrimination questionnaire (86) was the most widely used measure of racial discrimination, appearing in 19 studies in its original or adapted form (28, 34, 37–39, 41, 43–45, 47, 49, 54, 55, 57, 59, 60, 64, 69, 75), followed by individual items from the Pregnancy Risk Assessment Monitoring System (PRAMS) survey used in 16 studies (27, 30, 31, 33, 42, 46, 48, 52, 63, 66, 72, 77, 78, 81, 83, 85). Measurement across studies captured a broad range of exposure domains, including everyday interpersonal racism, major discriminatory events, healthcare-based mistreatment, gendered racism, and both direct and vicarious exposures, assessed across both past-year and lifetime timeframes.

**Figure 2:**
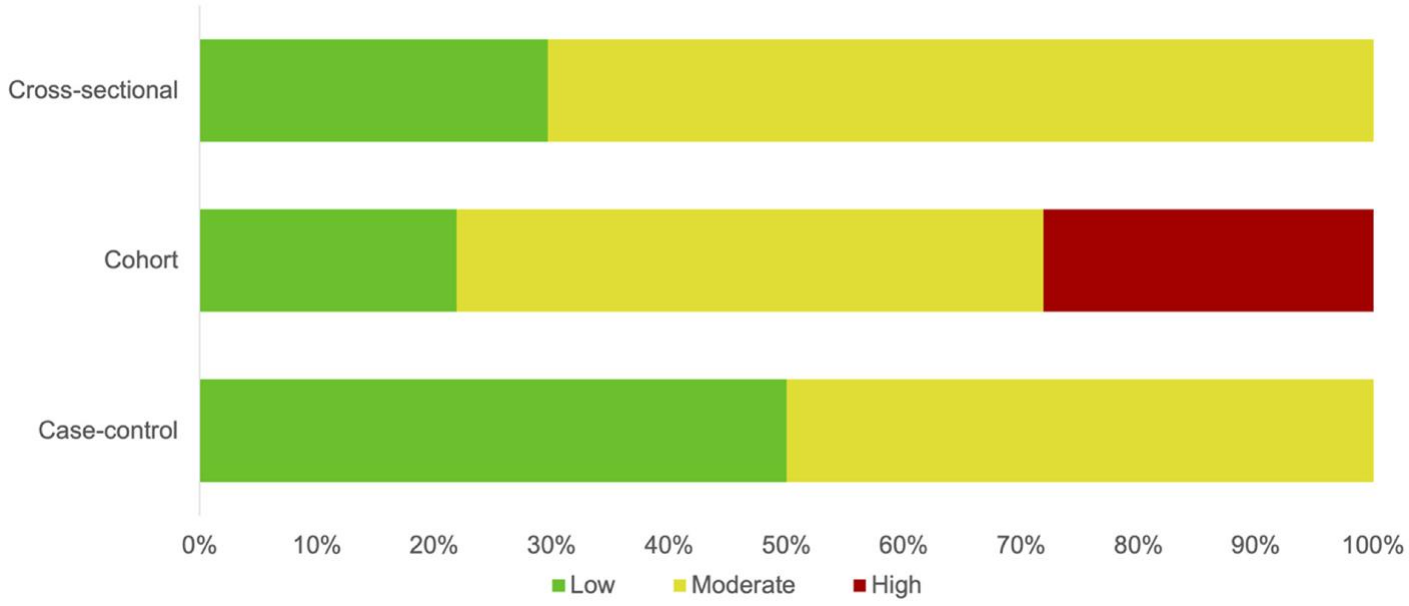
Summary of risk-of-bias assessments across included studies.

Figure 2 summarizes the distribution of risk of bias assessments across included studies. Most were rated as having moderate risk of bias, mainly due to limitations in sampling and incomplete adjustment for confounders. In contrast, measurement and reporting domains generally showed low risk, reflecting the use of validated instruments and standardized outcome definitions. Domain-level assessments by study design for individual studies are provided in Supplementary Figures S1–S3.

Studies included in the review examined a broad range of maternal and neonatal outcomes in relation to self-reported interpersonal racial discrimination. Maternal outcomes included HDP (54, 57, 63), GDM, (48, 54) mode of delivery (54, 81, 83), and PPD (29, 30, 33, 35, 41, 42, 46, 49–51, 58, 59, 66, 70, 72, 76–78, 84, 85). Neonatal outcomes included gestational duration –PTB (25–27, 31, 32, 35, 38–40, 43, 44, 47, 52, 53, 55, 57, 65, 67–69, 71, 73, 74, 79, 83) and gestational age (28, 45, 47, 54, 56, 62, 65, 82), and fetal growth indicators including birthweight, LBW, SGA, and LGA (27, 32, 34, 37, 41, 45, 47, 52, 56, 57, 60–62, 64, 65, 69, 73, 75, 80, 82, 83). No eligible studies reported infant mortality or NICU admission. Table 2 presents evidence direction plots summarizing the reported direction of associations across all included studies, including both adjusted and unadjusted analyses. For maternal outcomes, associations with PPD were generally consistent in direction, whereas HDP, GDM, and mode of delivery were largely null or mixed. For neonatal outcomes, findings for PTB, gestational age, and fetal growth outcomes were a mixture of positive and null directions.

**Table 2:** Direction of associations between self-reported racial discrimination and maternal and neonatal outcomes across all included studies.

Study	Study Design	Maternal				Neonatal					
		HDP	GDM	Mode of Delivery	PPD	PTB	Gestational Age	Birthweight	LBW	SGA	LGA
Ard et al., (26)	CSS	..	..	..	..	▴	..	..	..	..	..
Barber et al., (27)	CSS	..	..	..	..	◄►*, ▴**	..	..	▴*	◄► *	..
Barcelona et al., (28)	PCS	..	..	..	..	..	◄►	..	..	..	..
Becares et al., (29)	PCS	..	..	..	▴*	..	..	..	..	..	..
Bossick et al., (30)	CSS	..	..	..	▴	..	..	..	..	..	..
Bower et al., (31)	CSS	..	..	..	..	▴*	..	..	..	..	..
Brown et al., (32)	CSS	..	..	..	..	◄► *	..	..	◄► *	◄► *	◄►
Cabezas et al., (33)	CSS	..	..	..	▴	..	..	..	..	..	..
Christian et al., (34)	PCS	..	..	..	..	..	..	▾	..	..	..
Clarke et al., (35)	PCS	..	..	..	..	▴	..	..	..	..	..
Clarke et al., (36)	CSS	..	..	..	▴	..	..	..	..	..	..
Collins et al., (37)	CCS	..	..	..	.	.	.	..	◄► *	..	..
Collins et al., (38)	CCS	..	..	..	..	▴	..	..	▴*, ◄►**	..	..
Daniels et al., (39)	CSS	..	..	..	..	◄►	..	..	..	..	..
Davidson et al., (40)	ACS	..	..	..	..	◄►	..	..	..	..	..
Dixon et al., (41)	PCS	..	..	..	◄►	..	..	▾	..	..	..
Docherty et al., (42)	CSS	..	..	..	◄► *	..	..	..	..	..	..
Dole et al., (43)	PCS	..	..	..	..	◄► *	..	..	..	..	..
Dole et al., (44)	PCS	..	..	..	..	▴*	..	..	..	..	..
Dominguez et al., (45)	PCS	..	..	..	..	..	◄►	▾	..	..	..
Du et al., (46)	CSS	..	..	..	▴*	..	..	..	..	..	..
Eatman et al., (47)	PCS	..	..	..	..	◄► *	▾	◄►	..	◄► *	..
Erbetta et al., (48)	CSS	..	◄►	..	..	..	..	..	..	..	..
Ertel et al., (49)	PCS	..	..	..	◄► *	..	..	..	..	..	..
Ertel et al., (49)	PCS	..	..	..	◄► *	..	..	..	..	..	..
Flores-Rodriguez et al., (50)	CSS	..	..	..	▴*	..	..	..	..	..	..
Floyd James et al., (51)	CSS	..	..	..	▴	..	..	..	..	..	..
Fowlin et al., (52)	CSS	..	..	..	..	◄►	..	..	◄►	◄►	–
Fryer et al., (53)	RCS	..	..	..	..	◄►, ▴**	..	..	..	..	..
Gillespie et al., (54)	PCS	◄►	◄►	◄►	..	..	◄►	..	..	..	..
Giurgescu et al., (55)	CSS	..	..	..	..	◄► *	..	..	..	..	..
Green et al., (56)	PCS	..	..	..	..	..	◄►	..	◄►	..	..
Grobman et al., (57)	PCS	◄►	..	..	..	◄► *	..	..	..	◄► *	..
Harden et al., (58)	PCS	..	..	..	▴	..	..	..	..	..	..
Heldreth et al., (59)	CSS	..	..	..	▴	..	..	..	..	..	..
Hilmert et al., (60)	PCS	..	..	..	..	..	..	▾, ◄►**	..	..	..
Janevic et al., (61)	CSS	..	..	..	..	..	..	..	▴*	..	..
Korte et al., (62)	PCS	..	..	..	..	..	◄►	◄►	..	..	..
Lee et al., (63)	CSS	◄►	..	..	..	..	..	..	..	..	..
Lespinasse et al., (64)	CCS	..	..	..	..	..	..	..	▴	..	..
Liu et al., (65)	PCS	..	..	..	..	▴, ◄►**	▾, ◄►**	▾, ◄►**	◄►	..	..
Liu et al., (65)	PCS	..	..	..	..	▴	◄►	▾	▴	..	..
McDonald et al., (66)	CSS	..	..	..	◄► *	..	..	..	..	..	..
Mendez et al., (67)	RCS	..	..	..	..	◄► *	..	..	..	..	..
Misra et al., (68)	ACS	..	..	..	..	◄►	..	..	..	..	..
Mustillo et al., (69)	PCS	..	..	..	..	▴*, ◄►**	..	..	◄►	..	..
Njoroge et al., (70)	PCS	..	..	..	▴*	..	..	..	..	..	..
Rankin et al., (71)	CCS	..	..	..	..	▴	..	..	▴	..	..
Ringenary et al., (72)	CSS	..	..	..	▴*	..	..	..	..	..	..
Rosenberg et al., (73)	PCS	..	..	..	..	▴, ◄►**	..	..	◄►	..	..
Scholaske et al., (74)	RCS	..	..	..	..	▴*	..	..	..	..	..
Schuppie et al., (75)	CSS	..	..	..	..	..	..	..	◄►	..	..
Scroggins et al., (76)	RCS	..	..	..	▴	..	..	..	..	..	..
Segre et al., (77)	CSS	..	..	..	▴*	..	..	..	..	..	..
Shour et al., (78)	CSS	..	..	..	◄► *	..	..	..	..	..	..
Slaughter-Acey et al., (79)	PCS	..	..	..	..	▴	..	..	..	..	..
Slaughter-Acey et al., (80)	ACS	..	..	..	..	..	..	..	..	◄► *	..
Spinner et al., (81)	CSS	..	..	◄►	..	..	..	..	..	..	..
Thayer et al., (82)	PCS	….	..	..	..	..	▾	◄►	..	..	..
Thomas et al., (83)	CSS	..	..	▴	..	▴	..	..	▴	..	..
Walker et al., (84)	CSS	..	..	..	◄►	..	..	..	..	..	..
Weeks et al., (85)	CSS	..	..	..	▴*	..	..	..	..	..	..
Wheeler et al., (25)	PCS	..	..	..	..	◄► *	..	..	..	..	..

▴ = Positive association (higher odds/risk or higher value of outcome); ▾ = Negative association (lower odds/risk or lower value of outcome); ◄► = No significant or mixed results. CCS, case-control study; CSS, cross-sectional study; GDM, gestational diabetes mellitus; HDP, hypertensive disorders of pregnancy; LBW, low birth weight; LGA, large for gestational age; PCS, prospective cohort study; PPD, postpartum depression; PTB, preterm birth; RCS, retrospective cohort study; SGA, small for gestational age.

* Adjusted estimates included in meta-analysis.

** Estimates for two separate analyses in the study.

Adjustment strategies varied substantially across studies. Most analyses controlled for core sociodemographic variables, including maternal age, education, household income, and race or ethnicity. However, inclusion of clinical and behavioural covariates (e.g., smoking, alcohol use, body mass index, parity, and prior obstetric history) was inconsistent. Adjustment for psychosocial factors (e.g., depression history, stress, social support) and contextual exposures (e.g., neighbourhood deprivation, crime, or environmental conditions) was infrequent.

All studies evaluating HDP reported null associations (54, 57, 63). The two studies assessing GDM also found no evidence of an association (48, 54). Findings for mode of delivery were mixed: one study reported higher odds of caesarean section among women reporting interpersonal experiences of racial discrimination (83), whereas two reported no association (54, 81). These outcomes could not be pooled in meta-analysis because of methodological heterogeneity across studies.

**Figure 3:**
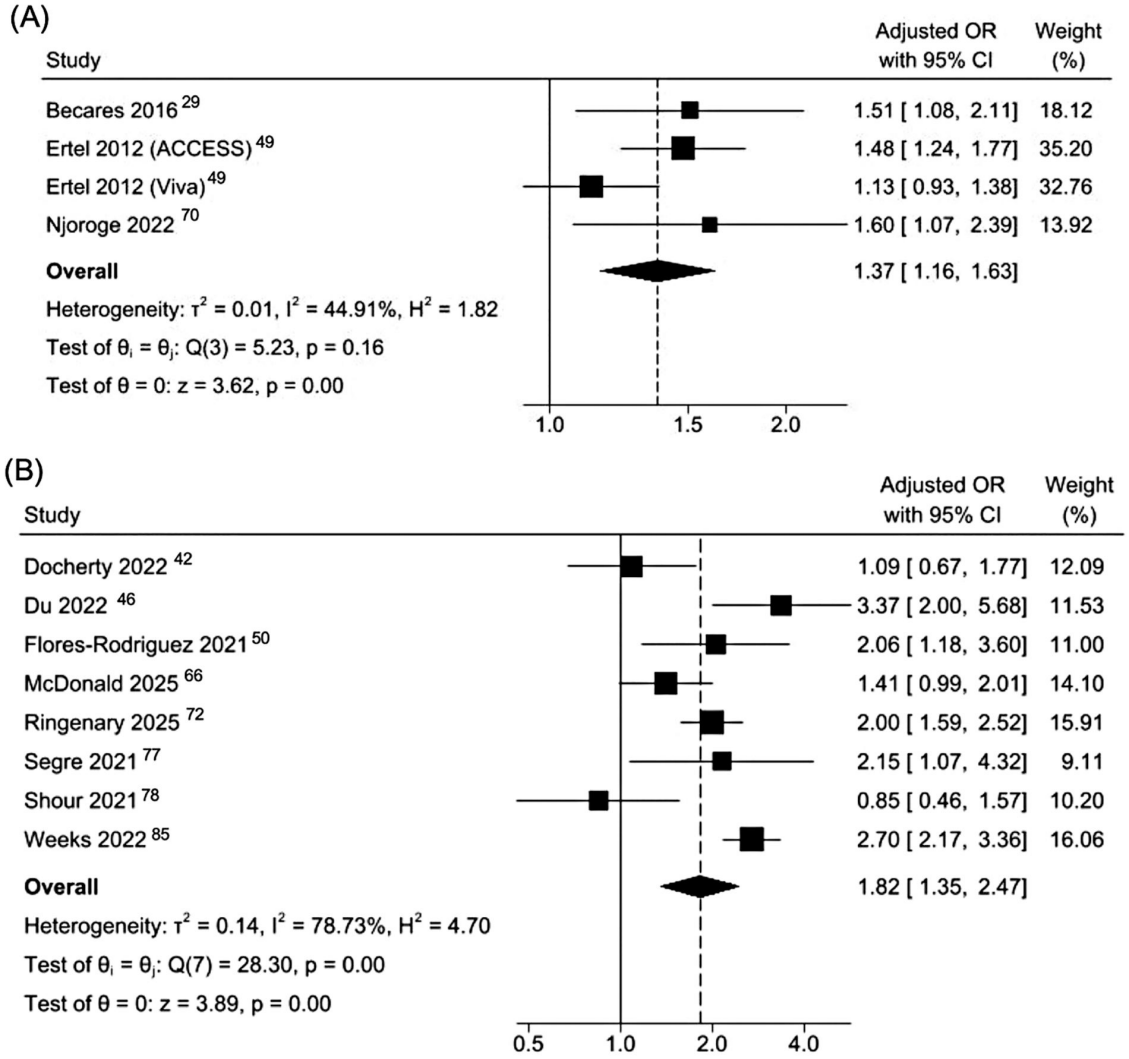
Meta-analyses of cohort and cross-sectional studies reporting adjusted odds ratios for the association between racial discrimination and postpartum depression. **(A)** Cohort studies. **(B)** Cross-sectional studies.

Evidence for PPD was more consistent. Most studies reported higher odds of PPD among women experiencing interpersonal racial discrimination. A meta-analysis of four cohort studies yielded a pooled aOR of 1·37 (95% CI 1·16–1·63; I^2^ = 44·9%) (Figure 3A). A meta-analysis of eight cross-sectional studies also indicated higher odds of PPD (pooled aOR 1·82 (95% CI 1·35–2·47), although heterogeneity was substantial (I^2^ = 78·7%) (Figure 3B).

**Figure 4:**
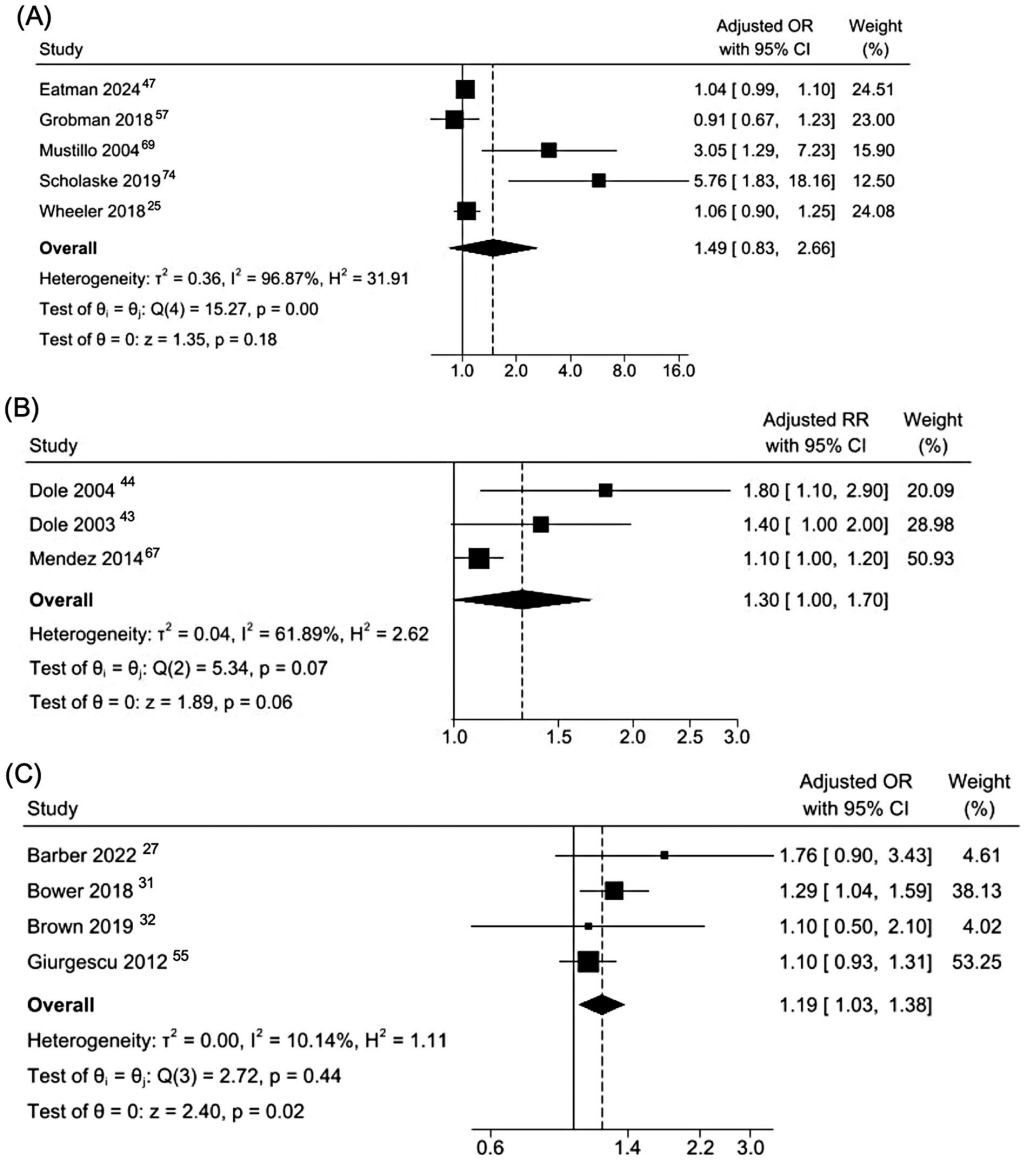
Meta-analyses of cohort and cross-sectional studies reporting adjusted associations between racial discrimination and preterm birth. **(A)** Cohort studies reporting adjusted odds ratios. **(B)** Cohort studies reporting adjusted risk ratios. **(C)** Cross-sectional studies reporting adjusted odds ratios.

Gestational outcomes were the most frequently examined neonatal outcome. A meta-analysis of five cohort studies found no association between self-reported interpersonal racial discrimination and PTB, although the estimate was highly heterogeneous (pooled aOR 1·49; 95% CI: 0·83–2·66, I^2^: 96·8%; Figure 4A). A separate meta-analysis pooling aRR from three cohort studies yielded a similar, not statistically significant estimate with substantial heterogeneity (pooled aRR 1·30; 95% CI 1·00–1·70; I² = 61·8%; Figure 4B). In contrast, a meta-analysis of four cross-sectional studies showed a small but statistically significant association with low between-study heterogeneity (pooled aOR 1·19; 95% CI 1·03–1·38; I² = 10·1%; Figure 4C).

Findings for gestational age at birth measured as a continuous outcome were inconsistent across prospective cohort studies. Four studies (28, 45, 47, 54) reported shorter a lower mean gestational age at delivery among women experiencing interpersonal racial discrimination, whereas five found no significant association. Meta-analysis was not feasible due to heterogeneity in racial discrimination measures and the types of β coefficients reporting gestational age (e.g., z-scores, days, or weeks).

Nineteen studies examined fetal growth, evaluating birthweight as a continuous variable, LBW (<2,500 g), or very LBW (<1,500 g). Among studies assessing continuous birthweight, six prospective cohorts reported lower birthweight associated with higher racial discrimination, whereas three reported no difference. Among studies evaluating LBW and very LBW, findings were mixed. Seven studies observed increased odds of LBW or very LBW among women reporting experiences of interpersonal racial discrimination in one or more domains, while five studies reported no association.

**Figure 5:**
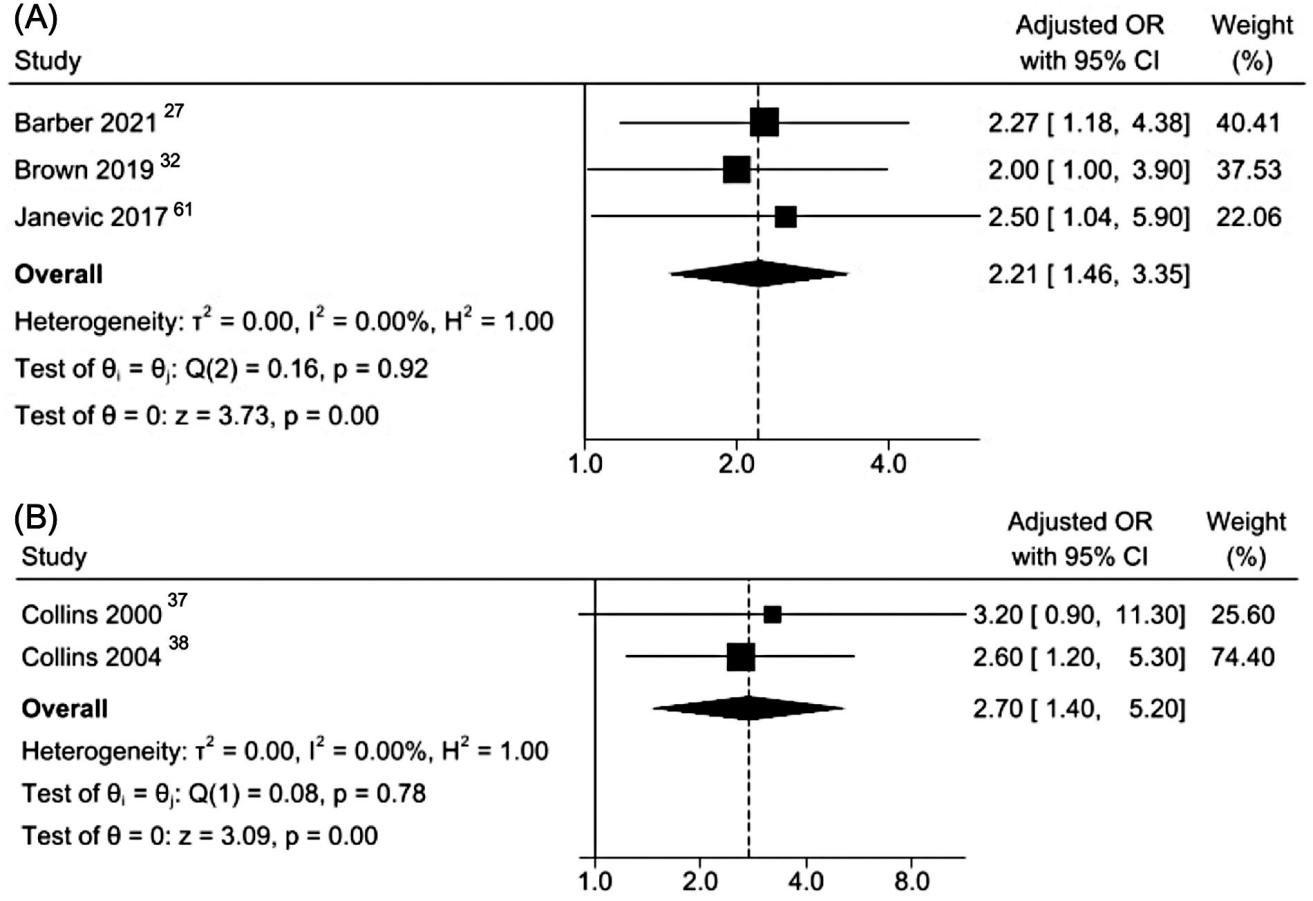
Meta-analyses of cross-sectional and case–control studies reporting adjusted odds ratios for the association between racial discrimination and low birth weight. **(A)** Cross-sectional; low birth weight (<2,500 g). **(B)** Case–control studies; very low birth weight (<1,500 g).

A meta-analysis of three cross-sectional studies examining LBW showed that women reporting interpersonal racial discrimination had over twice the odds of delivering a LBW infant compared to those who did not (pooled aOR 2·21, 95% CI 1·46–3·35; I^2^ = 0%; Figure 5A). Similarly, a meta-analysis of two case-control studies, yielded a pooled aOR of 2·70 (95% CI 1·40–5·20; I^2^ = 0%; Figure 5B); indicating increased likelihood of very LBW among women experiencing interpersonal racial discrimination.

**Figure 6:**
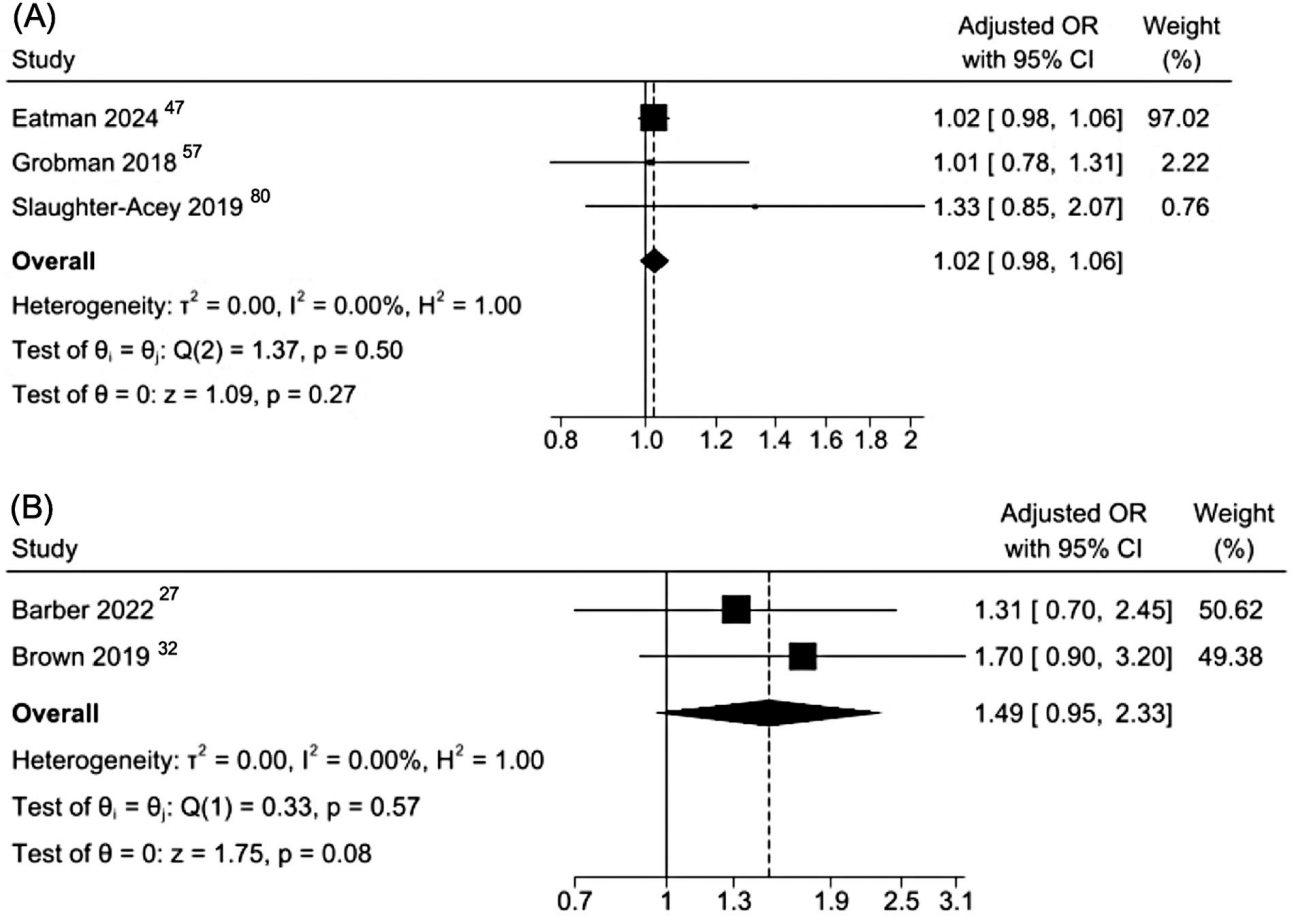
Meta-analysis of cohort and cross-sectional studies reporting adjusted odds ratios for the association between racial discrimination and small for gestational age. **(A)** Cohort studies. **(B)** Cross-sectional studies. The estimate from Slaughter-Acey et al, 2019) corresponds to the ≥25-year maternal age group to align with the mean age of participants in the other two cohort studies.

In a meta-analysis of data from three cohort studies on adult pregnancies (47, 57, 80), past-year personal experiences of interpersonal racial discrimination were not significantly associated with increased odds of SGA (pooled aOR 1·02, 95% CI 0·98–1·06; I^2^ = 0%; Figure 6A). These results were similar when data from two cross-sectional studies (27, 32) were combined in a meta-analysis: there were no statistically significant association between self-reported interpersonal racial discrimination and SGA (pooled aOR 1·49, 95% CI 0·95–2·33; I^2^ = 0%; Figure 6B).

The association between maternal exposure to prenatal care-perceived interpersonal racial discrimination and infants born LGA was examined in a single cross-sectional study (32) conducted among Aboriginal and Torres Strait Islanders in South Australia. No significant association was observed.

No studies examined the impact of racial discrimination on infant mortality and NICU admissions.

## 4 Discussion

This systematic review and meta-analyses synthesizes over three decades of research on self-reported interpersonal racial discrimination and maternal and neonatal health. Across 63 included studies involving over 1·4 million women, the most consistent association was between experiences of interpersonal racial discrimination and PPD. In contrast, evidence for most maternal outcomes in the review, including HDP, GDM and mode of delivery, was limited or inconsistent. For neonatal outcomes, results varied by study design and outcome. Associations were observed most consistently for LBW and very LBW, particularly in cross-sectional and case-control studies, whereas findings for PTB and SGA were less conclusive and heterogeneous.

Our findings both align with and diverge from previous reviews (11–16). Consistent with earlier syntheses, we found support for an association between self-reported interpersonal racial discrimination and LBW. In contrast, prior reviews reported more consistent evidence for PTB, whereas our design-stratified meta-analyses of adjusted estimates showed less consistent associations for this outcome, particularly in cohort studies. Earlier reviews did not examine PPD, which emerged as the most consistent association in this review. These discrepancies likely reflect differences in analytical strategies, including restrictions to adjusted estimates, stratification by study design, and inclusion of more recent studies in this review.

The association with PPD was robust across study designs and meta-analyses. This is not surprising given the sensitivity of mood disorders to chronic psychosocial stressors (87). Experiences of interpersonal racial discrimination may contribute through prolonged stress, social isolation, and strained social relationships during pregnancy and the postpartum period. The consistency of this association across study designs suggests that interpersonal racial discrimination is an important and underrecognized contributor to maternal mental health.

Evidence for neonatal outcomes showed a heterogeneous pattern. For PTB, pooled estimates from cohort studies did not show a clear association, whereas meta-analysis of cross-sectional studies with low heterogeneity suggested a modest association. Given the stronger temporality in cohort designs, confidence in an association with PTB is limited, and the cross-sectional results should be interpreted cautiously. For SGA, we found no consistent evidence of an association in either cohort or cross-sectional analyses. Differences in study design, exposure measurement, and residual confounding, likely contribute to much of the heterogeneity in the results. Residual confounding may also reflect variability in covariate adjustment across studies. Although we pooled adjusted estimates, models differed in whether they accounted for psychosocial factors (e.g., baseline mental health, stress, social support) and behavioral/clinical factors (e.g., smoking, BMI, parity, obstetric history), which may have contributed to heterogeneity and potential residual bias. Reporting of associations with gestational age and fetal growth varied widely, precluding a meaningful synthesis. In contrast, results for LBW and very LBW were more consistent, suggesting that interpersonal racial discrimination may affect fetal growth through pathways not fully reflected in gestational age alone, and potentially involving maternal stress, placental dysfunction, metabolic dysregulation, and inflammatory processes during pregnancy.

Substantial heterogeneity in exposure measurement may have contributed to inconsistent findings for neonatal outcomes. Racial discrimination was assessed as everyday interpersonal mistreatment, major discriminatory events, healthcare-based racism, emotionally distressing experiences, or lifetime exposure. These approaches may capture qualitatively different exposure constructs –acute or time-limited events vs. chronic, cumulative experiences– with potentially different biologic pathways and latency periods relevant to specific perinatal outcomes, including differences in recall periods (lifetime vs. pregnancy/postpartum). Such differences in conceptualization and temporal framing may have limited comparability across studies and attenuated pooled estimates, particularly for outcomes such as PTB and SGA that may depend on chronic or cumulative stress pathways rather than short-term exposures alone. This heterogeneity also limits the generalizability of the pooled estimates.

The findings have important clinical and policy implications. The consistent association between perceived interpersonal racial discrimination and PPD highlights the need for routine assessment of social stressors in perinatal care, alongside mental health screening during pregnancy and the postpartum period. Current clinical guidelines rarely address racial discrimination explicitly as a risk factor, yet the magnitude and consistency of this association suggest it warrants systematic attention. At the policy level, these findings reinforce the need for structural interventions that address discrimination as a public health issue, rather than framing it solely as an individual experience.

The geographic concentration of evidence in the USA limits global generalizability; nonetheless, the direction of associations for several maternal and neonatal outcomes observed in studies from other settings suggests relevance beyond the USA. Most studies focused on non-Hispanic Black women in the USA, with relatively few examining Indigenous or racialized populations outside high-income countries. This narrow distribution of the evidence limits generalizability and highlights a major knowledge gap, emphasizing the need for research in diverse sociopolitical environments where the nature, expression, and consequences of racial discrimination may differ. The absence of studies examining infant mortality and NICU admissions is also notable, given the clinical importance of these outcomes.

Future research should prioritize longitudinal designs, improve standardization and validation of perceived interpersonal racial discrimination measures across settings, and extend investigation to outcomes that remain understudied, including infant mortality and NICU admission. Research in non-USA and low- and middle-income settings is particularly needed to understand how health consequences of racial discrimination vary across sociopolitical contexts. Studies should also further examine biological mechanisms linking psychosocial stress to maternal and neonatal health outcomes. Existing conceptual frameworks suggest mediating pathways including hypothalamic–pituitary–adrenal axis activation and cortisol dysregulation, inflammatory and immune changes (88), and stress-related behavioural pathways (e.g., sleep disruption, substance use, or reduced engagement with antenatal care) that may be linked to placental function and fetal growth. Future research should test these mediators and examine moderators of risk and resilience across diverse racialized populations globally.

### 4.1 Strengths and limitations

This review has several strengths. It provides the most up-to-date quantitative synthesis of self-reported interpersonal racial discrimination and perinatal health, with nearly 40% of included studies published since 2021, reflecting the growing recognition of racial discrimination as a social determinant of perinatal health. The review also considers a wider range of outcomes than previous syntheses and incorporates multiple study designs, allowing comparisons of findings across analytic approaches and identifying areas of consistency and uncertainty.

The findings must be interpreted with caution in light of several limitations. Because interpersonal racial discrimination can only be assessed by self-report, variability in measurement instruments and recall periods likely contributed to exposure misclassification across studies (89). In addition, the literature did not support instrument-specific synthesis; too few studies used the same discrimination measure within each outcome to allow meaningful subgroup meta-analyses. Residual confounding is also likely; differences in covariate adjustment, particularly inconsistent control for behavioural, clinical, and psychosocial variables, likely contributed to heterogeneity and may partially explain inconsistencies in observed associations. Differences in covariate adjustment across studies further limit comparability and may affect the generalizability of the pooled estimates. Meta-analysis was not feasible for several studies and outcomes due to heterogeneity in definitions. Adolescent populations were rarely analyzed separately, and evidence remains sparse outside high-income settings.

## 5 Conclusions

This review provides consistent evidence that self-reported interpersonal racial discrimination is associated with PPD and LBW, with more variable evidence for PTB and other perinatal outcomes. However, because most included studies were conducted in the United States, the generalizability of these findings to other sociopolitical and health-system contexts may be limited. These findings support the inclusion of social stressors such as interpersonal racial discrimination as an important and modifiable determinant of maternal and child health. Improving measurement, increasing geographic diversity, and better integrating social exposures into perinatal research and care are priorities in the field. Strengthening this evidence base will clarify where interventions are most needed to reduce inequities in maternal and neonatal outcomes.

## Data Availability

The original contributions presented in the study are included in the article/Supplementary Material, further inquiries can be directed to the corresponding author.
