# Supplementary material for: Self-reported experiences of interpersonal racial discrimination and maternal and neonatal health: a systematic review and meta-analysis
Source: Front Reprod Health. 2026 Mar 16;8:1783126. doi: 10.3389/frph.2026.1783126 (PMC13033699; doi:10.3389/frph.2026.1783126)
Supplement: Supplementary file 1 [file Table1.docx]

Supplementary Material

**Supplementary Materials**

**Table S1:** Full search terms for Medline

Ovid MEDLINE(R) ALL <1946 to October 15, 2025>

|  | exp Pregnancy/ or exp Pregnant Women/ or exp Labor, Obstetric/ or exp Prenatal Care/ or (obstetric* or prenatal* or pregnan* or gestat* or perinatal or antenatal or parturi* or neonat*).mp. or (pregnan* or birth or childbirth or obstetric*).mp. |
| --- | --- |
|  | racism.mp. or exp Racism/ or racial disparities.mp. or ((race or racial) adj6 discrim*).mp. or exp Prejudice/ or prejud*.mp. or ((race or racial) adj6 bias).mp. or "unfair treat*".mp. or ((race or racial) adj6 oppress*).mp. or microaggression*.mp. |
|  | exp Delivery, Obstetric/ or exp Obstetric Labor/ or childbirth.mp. or (labo?r adj3 delivery).mp. or (mode of delivery or type of delivery).mp. or (labor or labour).mp. |
|  | exp Cesarean Section/ or (Caesarean or cesarean or cesarian).mp. |
|  | (((spontaneous or induc* or onset or length) adj3 (labor or labour)) or (episiotom* or vaginal delivery)).mp. |
|  | exp Obstetric Labor Complications/ or exp Extraction, Obstetrical/ or (((normal or instrumental or assisted) adj2 (delivery or birth or childbirth)) or (forceps or ventouse or vacuum extraction)).mp. |
|  | exp Diabetes, Gestational/ or diabet*.mp. or exp Diabetes Mellitus, Type 2/ or exp Diabetes Mellitus, Type 1/ or exp Blood Glucose/ or diabet*.mp. or exp Diabetes Mellitus/ or "Gestational Diabetes".mp. |
|  | hypertens*.mp. or exp Hypertension/ or exp Hypertension, Pregnancy-Induced/ or "Gestational Hypertens*".mp. |
|  | (pre-eclamp* or preeclamp* or "pre eclampsia" or toxemia* or toxaemia* or gestosis).mp. or exp Pre-eclampsia/ |
|  | (("post partum" or post-partum or postpartum or postnatal or post-natal or "post natal") adj3 (depress* or mood or mental health or depressive disorder*)).mp. or exp Depression, Postpartum/ |
|  | exp Pregnancy Outcome/ or pregnanc* outcom*.mp. or exp Pregnancy Complications/ or "maternal outcomes".mp. or exp Pregnancy Complications, Infectious/ or ((birth or pregnancy or childbirth) adj3 (outcome* or complication*)).mp. |
|  | 3 or 4 or 5 or 6 or 7 or 8 or 9 or 10 or 11 |
|  | exp Obstetric Labor, Premature/ or exp Premature Birth/ or exp Infant, Premature/ or preterm.mp. or premature.mp. or Gestational Age.mp. or exp Gestational Age/ or "preterm delivery".mp. or preterm birth.mp. or exp Infant, Premature, Diseases/ |
|  | low birth weight.mp. or exp Infant, Low Birth Weight/ |
|  | neonat* mortality.mp. or exp Infant Mortality/ or ((perinatal or fetal or fetus or foetal or foetus or neonat* or newborn* or infant*) adj3 (mortality or morbidity or death or outcome* or complication*)).mp. |
|  | exp Intensive Care Units, Neonatal/ or exp Intensive Care, Neonatal/ or nicu*.mp. or ncu*.mp. or ((neonatolog* or nurser* or neonat* or newborn*) adj6 (ward* or department* or unit* or icu* or "intensive care")).mp. |
|  | 13 or 14 or 15 or 16 |
|  | 12 or 17 |
|  | 1 and 2 and 18 |

**Figure S1.** Study-level risk-of-bias assessments for cohort studies using the Newcastle–Ottawa Scale


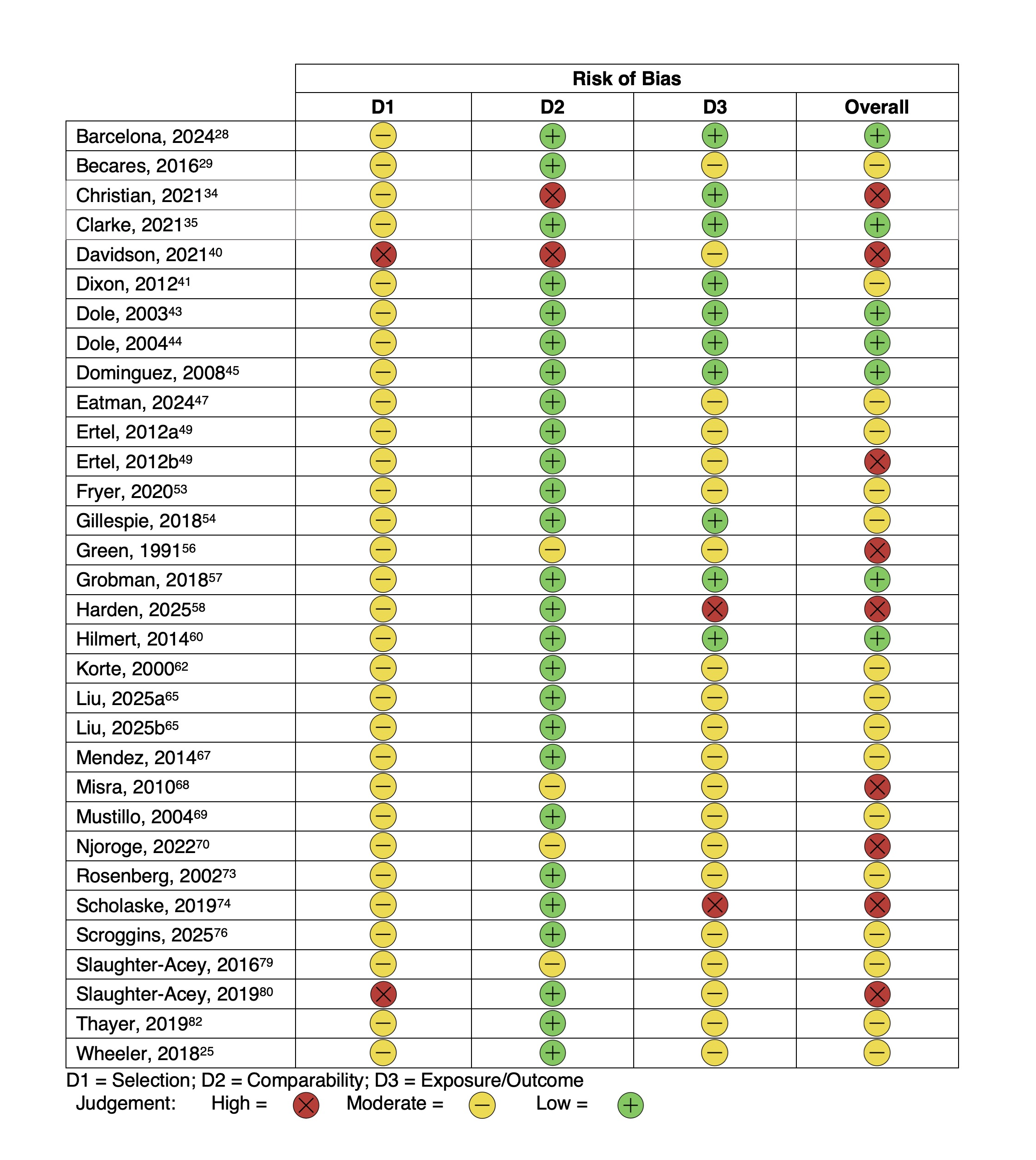


**Figure S2.** Study-level risk-of-bias assessments for case–control studies using the Newcastle–Ottawa Scale

**
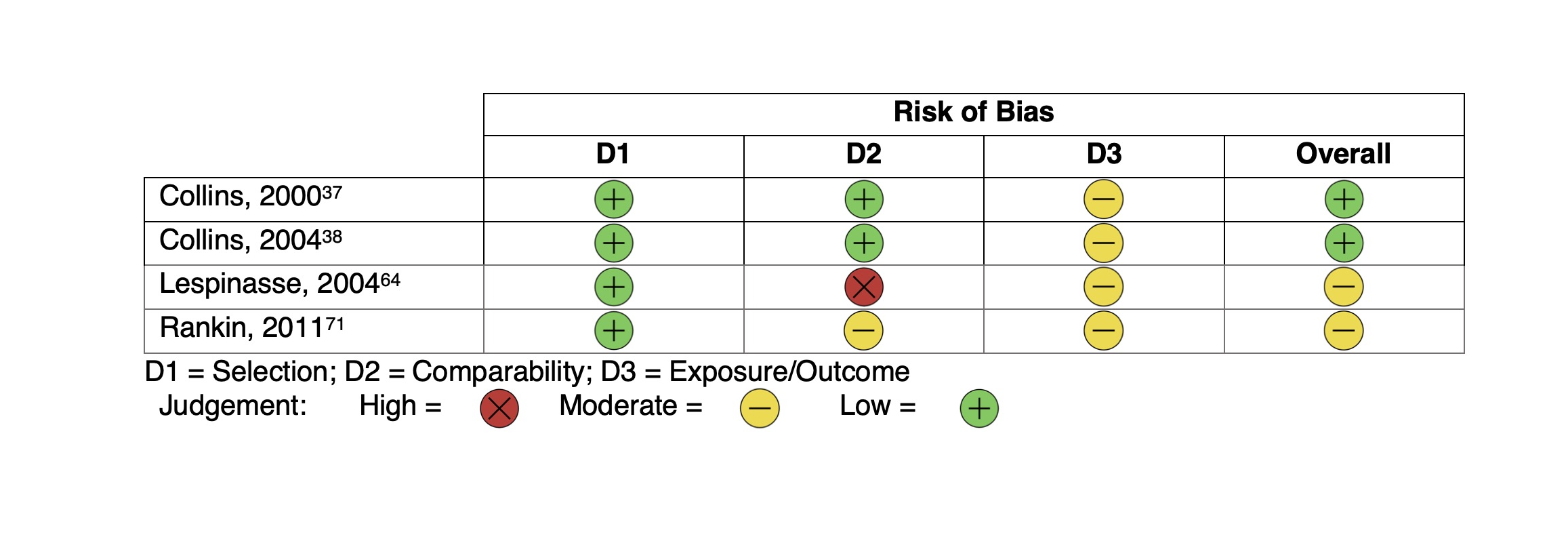
**

**Figure S3.** Study-level risk-of-bias assessments for cross-sectional studies using the Appraisal Tool for Cross-Sectional Studies (AXIS)

**
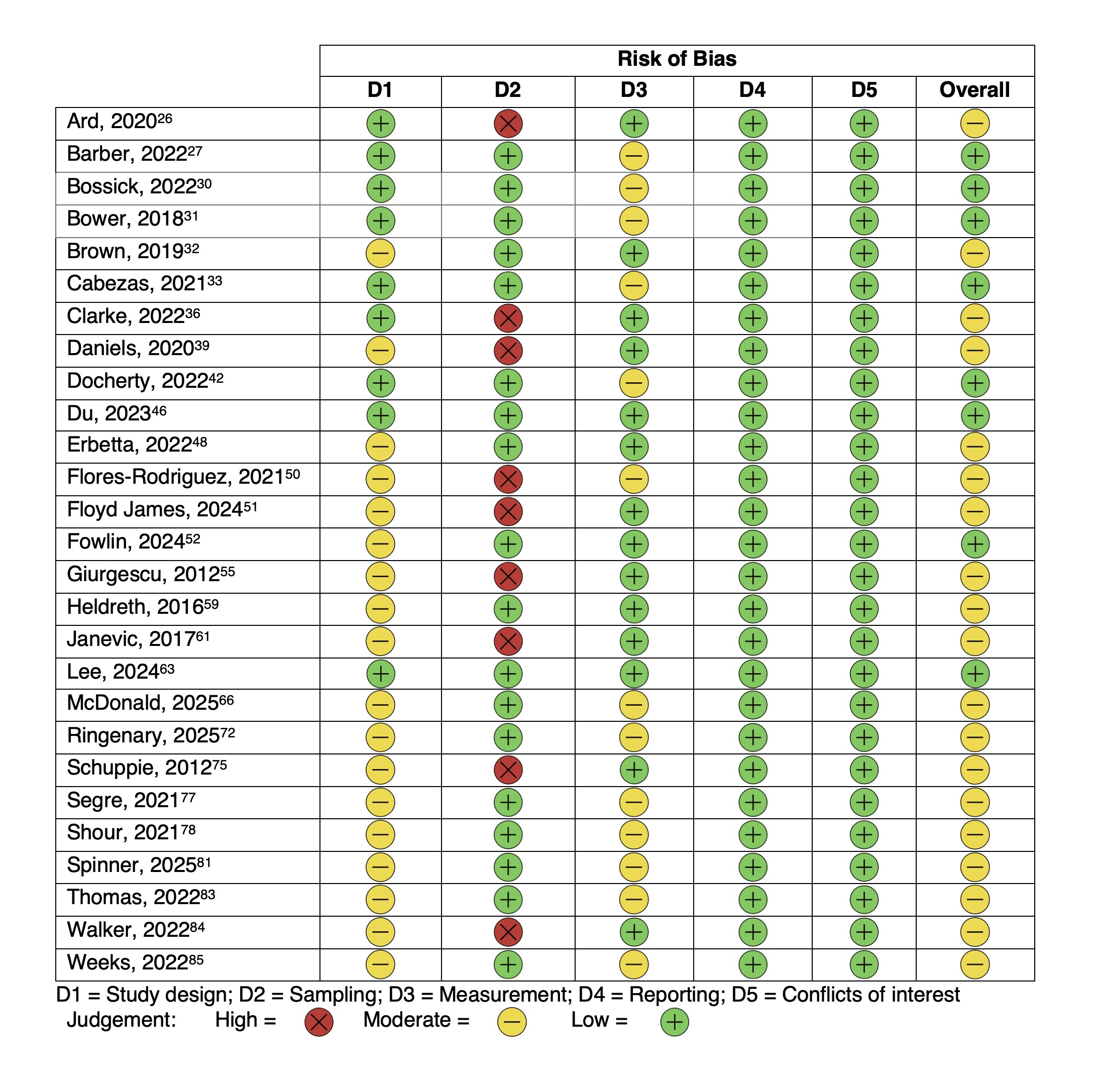
**
